# Synergistic activity of rifampicin and polymyxin B against intracellular Gram-negative ESKAPE pathogens involves bacterial membrane alterations and enhanced oxidative damages

**DOI:** 10.1128/aac.01319-25

**Published:** 2025-11-28

**Authors:** Vallo Varik, Gang Wang, George Kritikos, Manuel Banzhaf, Emilien Drouot, Alexandra Koumoutsi, Françoise Van Bambeke

**Affiliations:** 1 Pharmacologie cellulaire et moléculaire, Louvain Drug Research Institute, Université catholique de Louvain 508610 , Brussels, Belgium; 2 European Molecular Biology Laboratory 9471, Heidelberg, Germany; 3 Newcastle University Biosciences Institute, Faculty of Medical Sciences, Newcastle University 5994, Newcastle upon Tyne, United Kingdom; Providence Portland Medical Center, Portland, Oregon, USA

**Keywords:** drug-drug interaction, rifampicin, polymyxin B, Gram-negative bacteria, ESKAPE pathogens, drug repurposing, intracellular infection

## Abstract

Antibiotic-resistant bacteria, particularly the ESKAPE (*Enterococcus faecium, Staphylococcus aureus, Klebsiella pneumoniae, Acinetobacter baumannii, Pseudomonas aeruginosa,* and *Enterobacter* spp.) pathogens, pose a major public health threat. Their ability to reside inside cells contributes to their persistence and resistance. Combining rifampicin with polymyxins is a much-discussed approach against multidrug-resistant Gram-negative bacterial infections. We therefore evaluated a combination of polymyxin B and rifampicin against Gram-negative clinical isolates in extracellular and intracellular *in vitro* models of infection. The combination was synergistic against intra- and extra-cellular forms of *P. aeruginosa, A. baumannii, E. coli*, and *K. pneumoniae*. This synergy was enhanced in an acidic environment resembling the host vacuole where intracellular bacteria reside. The combination remained synergistic against rifampicin and polymyxin B-resistant *P. aeruginosa*. To reveal the molecular underpinnings of the synergy, we used reverse genetics to identify and describe *P. aeruginosa* mutants more susceptible to the combination. They show altered membrane properties and more pronounced oxidative damage when exposed to the combination. This work sheds a new light on the mechanisms of the synergy between rifampicin and polymyxins, demonstrates its applicability to Gram-negative ESKAPE pathogens, including when residing intracellularly. Overall, the data suggest that repurposing rifampicin with polymyxin B can effectively target hard-to-eradicate intracellular bacteria.

## INTRODUCTION

Antimicrobial resistance (AMR) outpaces the development of new antimicrobials and threatens many aspects of modern medicine. In 2018, the World Health Organization issued a list of pathogens, colloquially known as ESKAPE (*Enterococcus faecium*, *Staphylococcus aureus*, *Klebsiella pneumoniae*, *Acinetobacter baumannii*, *Pseudomonas aeruginosa*, and *Enterobacter* species) pathogens, for which new treatments are urgently needed (1). ESKAPE pathogens are an important cause of hospital-acquired infections, with frequent isolates showing multiple drug resistance.

In addition to AMR, bacteria can escape the action of antibiotics and the immune system by hiding inside host cells and forming biofilms. The importance of an intracellular niche is increasingly appreciated for some classical extracellular bacteria such as *P. aeruginosa*, *K. pneumoniae*, *Escherichia coli,* and *S. aureus* (2, 3). Among them, *P. aeruginosa* is responsible for 10% of hospital-acquired infections (2), especially in intensive care units (4). It is notoriously hard to treat as, besides acquired resistance, *P. aeruginosa* is intrinsically resistant to many common antibiotics (5). Moreover, *P. aeruginosa* has been reported to reside intracellularly *in vitro* in phagocytic (6, 7) and epithelial (8–11) cells, as well as in infected mice (12) and patients (13).

To tackle hard-to-treat infections, drug combinations have multiple attractive properties. First, they address the urgency—if drugs in combination are approved for human use, it greatly facilitates the transfer from bench to bedside. Second, they surpass and alleviate the scarcity of new chemical scaffolds because new qualitative and quantitative properties emerge from drug combinations. The most beneficial quantitative property of combinations is synergy, that is, the activity of the mixture is greater than expected from the effects of each drug given alone. Synergy also expands the chemotherapy solution space by increasing therapeutic activity, enabling lower dosage, and mitigating the side effects of otherwise unfit monotherapies. Third, combinations can curtail the emergence of AMR by making it harder to evolve resistance (14). Furthermore, as drug interactions could be species-specific (15), they can be used to lessen the selective pressure for AMR and spare the host microbiome. Whether drug combinations have beneficial properties against intracellular forms of bacteria is not well known.

Under the Joint Programming Initiative on Antimicrobial Resistance (JPIAMR) framework (16), we set out to delineate the potential of misused and neglected antibiotics to treat Gram-negative infections (17). Preliminary screenings on various clinical bacterial isolates suggested the utility of the combination of rifampicin and polymyxin B (17–19). This combination has been evaluated in the clinics as a promising salvage therapy for multidrug-resistant infections (20–22). Rifampicin is a polyketide antibiotic that targets bacterial transcription and is an essential drug in combination against mycobacterial infections (23). Rifampicin has gained considerable attention for its possible interplay with other medications (24). Although antibiotics that accumulate well in bacterial cells are typically amphiphilic (25), rifampicin is large, hydrophobic, and thus poorly permeable in Gram-negative bacteria. However, its uptake is facilitated by membrane-permeabilizing cationic agents such as polymyxins (26). Polymyxin B is a polypeptide antibiotic targeting the outer membrane of Gram-negative bacteria (27).

Based on these premises, the aim of this study was to investigate whether synergy between rifampicin and polymyxin B also extends to intracellular forms of infection by *P. aeruginosa* and other Gram-negative ESKAPE pathogens and explore the possible underlying mechanisms.

In brief, we found that the rifampicin-polymyxin B combination is synergistic against intracellular forms of a wide range of ESKAPE bacteria (*P. aeruginosa*, *A. baumannii, E. coli,* and *K. pneumoniae*). Contrasting the results from intracellular infection models and laboratory growth medium, we revealed the synergy-promoting role of the acidic pH prevailing in intracellular compartments. To account for the synergy in molecular terms, we identified and characterized *P. aeruginosa* mutants with altered membrane properties and pronounced oxidative damage when exposed to the combination.

## RESULTS

### The rifampicin-polymyxin B combination is synergistic against intracellular *P. aeruginosa*

**Fig 1:**
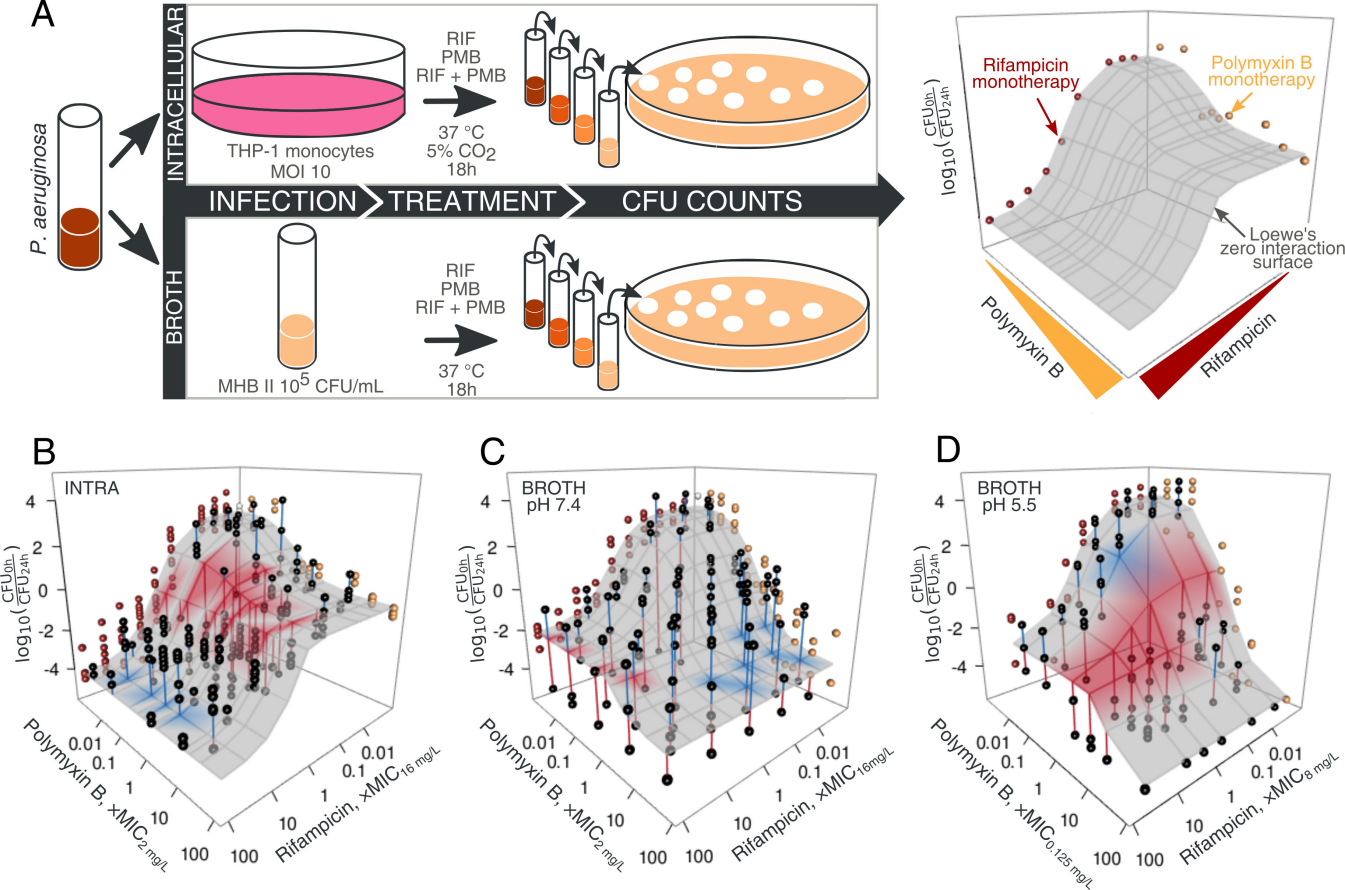
Promoted by an acidic environment, the rifampicin-polymyxin B combination synergizes against intracellular *P. aeruginosa*. (**A**) *P. aeruginosa* ATCC27853 was treated with rifampicin, polymyxin B, or their combination in infected monocytes or broth (cation-adjusted Mueller Hinton, MHBII). Residual CFU were counted after serial dilutions of the samples. A four-parameter concentration-response curve was fitted to the monotherapies to estimate pharmacodynamic parameters: *E_min_* (carrying capacity of the condition), *E_max_* (efficacy, i.e., maximal drug response), *EC_50_* (the concentration producing 50% of the maximal response), and *C_s_* (the concentration resulting in no net increase in bacterial numbers). Finally, the expected effect of the combination was calculated under Loewe’s null hypothesis across the concentration space. (**B**) The experimentally measured outcome of the two-drug combination on intracellular *P. aeruginosa* is shown with black circles (each circle represents a biological replicate of experiments performed in three technical replicates). The red lines signify synergy, that is, connect points to the surface if the measured CFU are below the expectation surface. Conversely, blue indicates antagonism. The surface was colored if the difference from expectation was statistically significant (*P* < 0.05 from a bootstrapped approximation of the expected result under Loewe’s null). (**C**) Monotherapies and combination results, along with the no-interaction expectation surface, for the results in MHBII broth at pH 7.4. (**D**) Same as (**C**), but with broth pH adjusted to 5.5 using 100 mM MES. On (**B**), (**C**), and (**D**), the concentration on the x- and the y-axis is expressed in multiples of measured MICs; the subscript in the axis title indicates the determined MIC for the condition (neutral broth [**B and C**] or broth with pH 5.5 [**D**]).

Working first with the *P. aeruginosa* reference strain ATCC 27853 and using the broth microdilution method to determine the lowest concentration of no turbidity, we measured the minimal inhibitory concentration (MIC) of rifampicin and polymyxin B to be 16 and 2 µg/mL, respectively. Using the MIC to orient ourselves in the concentration space, we then resolved each drug’s extra- and intra-cellular pharmacodynamics alone or in combination by following colony-forming units (CFU). In particular, we estimated the concentration-dependent effect of drugs after 24 h (i) in the intracellular infection model (6) and (ii) in cation-adjusted Mueller-Hinton broth (Fig. 1A). Assuming a shared *E_min_* (carrying capacity of the condition) and fixing slope at 1, we fit a four-parametric concentration-response curve to the monotherapies to estimate the efficacy (*E_max_,* maximal drug response) and potency (*EC_50_*, the inflection point of the four-parametric Hill curve, that is, the concentration producing 50% of the maximal response) of a given drug (Fig. 1A). Finally, we used the Loewe model to quantify the interaction of two drugs in combination (28). Loewe’s model assumes dose additivity, calculating the expected response as if the two drugs are the same. This approach was preferred to the widely used fractional inhibitory concentration index (FICI) method, which is particularly prone to reproducibility problems and influenced by the way concentrations are selected to calculate interactions (29).

**Fig 2:**
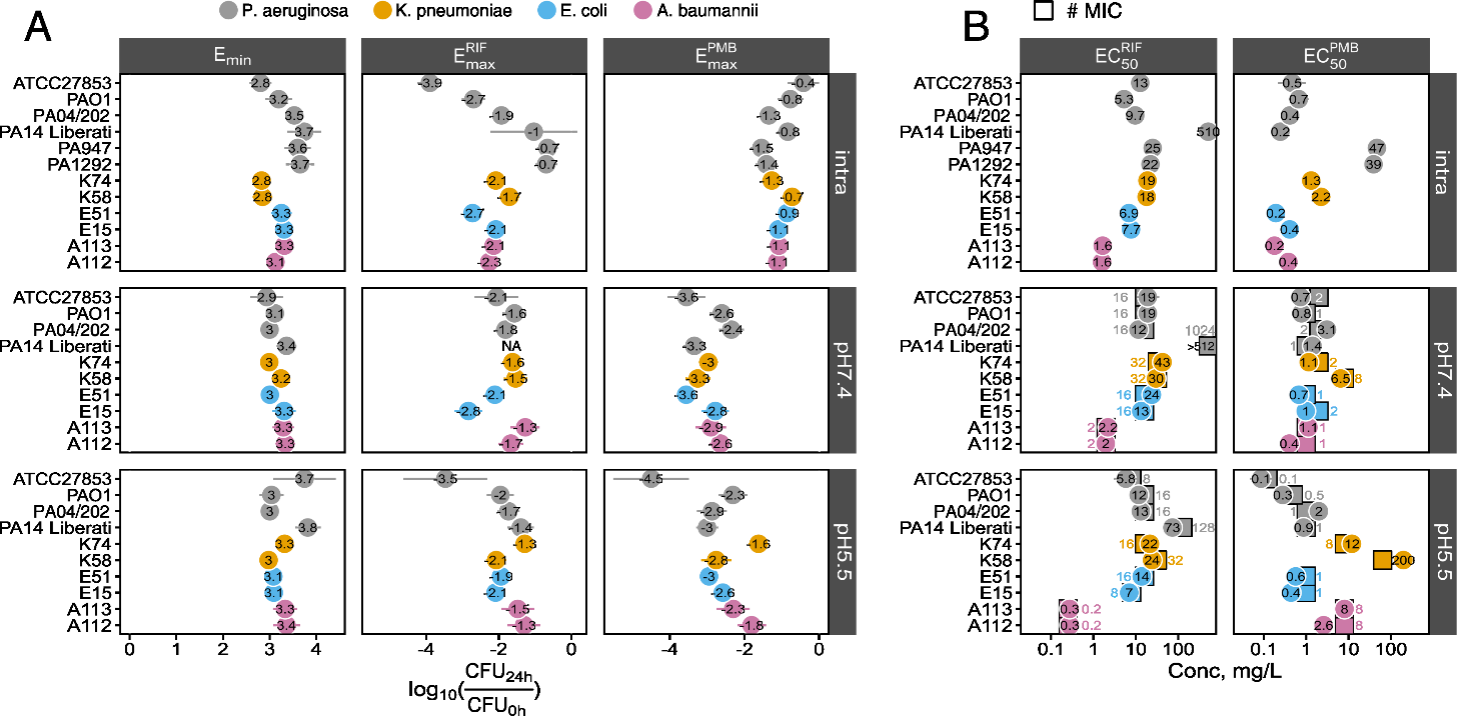
Pharmacodynamic parameters of monotherapies. (**A**) Carrying capacity (maximum yield in CFU/mL, *E_min_*) and maximum efficacy (*E_max_*) of rifampicin and polymyxin B (expressed as a logarithm of the change in CFU/mL after 24 h of treatment) are shown for a given strain (y-axis) and condition (facet). PA14 Liberati strain was too resistant to rifampicin in neutral broth (pH 7.4) to determine its *E_max_* with reasonable confidence and is therefore NA. (**B**) Drug potency, that is, half-maximal response (*EC_50_*, denoted by circles) of rifampicin and polymyxin B shown alongside MIC (squares). Error bars are 95% confidence intervals. *E_min_*, *E_max_*, and *EC_50_* were estimated using a four-parametric model with a shared *E_min_* per strain in a given condition and slope fixed at 1. MIC was measured by broth microdilution. ATCC27853, PAO1: common laboratory strains of *P. aeruginosa*; PA14 Liberati: PA14 of the transposon library (31); PA04/202: susceptible clinical isolate; PA947 and PA1292: polymyxin-resistant clinical isolates of *P. aeruginosa*; K74, K58, E51, E15, A113, and A112: two clinical isolates of *K. pneumoniae*, *E. coli*, and *A. baumannii*, respectively. All experiments have been performed at least three times in triplicates.

When used alone, in line with previous observations of attenuated intracellular activity for a wide variety of antibiotics (6, 30), polymyxin B exhibited three orders of magnitude higher maximal efficacy in broth (MHBII, pH 7.4) compared with intracellular conditions (Fig. 1B vs Fig. 1C, 2A; Fig. S1B). In contrast, rifampicin had two orders of magnitude lower efficacy in broth (MHBII, pH 7.4) compared with intracellular bacteria (Fig. 1B vs C and 2A; Fig. S1A). At the same time, the potency of both drugs (quantified as *EC_50_*) remained unaltered and close to their broth MICs across both environments (Fig. 2B).

The combination of rifampicin and polymyxin B was synergistic against intracellular forms of *P. aeruginosa*, most notably at the concentration of 0.3 times the MIC of rifampicin (Fig. 1B). In stark contrast, this interaction disappeared in broth (MHBII, pH 7.4; Fig. 1C).

Since intracellular *P. aeruginosa* is located in the vacuoles (6), we hypothesized that the low pH might improve the efficacy of rifampicin and increase the synergy of the combination. To test this idea, we attempted to acidify the broth using HCl. Contrary to the common notion of environmental acidification by various microorganisms (32), *P. aeruginosa* neutralized the acidified broth within 9 h (Fig. S1G).

Upon buffered acidification of the medium (pH 5.5 by 100 mM MES) to maintain the acidic pH throughout the experiment (Fig. S1G), (i) the concentration-response curve of rifampicin became superimposable to that observed in the intracellular environment (Fig. S1A), (ii) the maximal efficacy of rifampicin increased by 1.5 orders of magnitude (Fig. 2A; Fig. S1A), and (iii) synergy was re-established (Fig. 1C vs D), although curiously centered around the MIC of polymyxin B. In addition, observing the kinetics of bacterial killing in time, intracellular and broth at pH 5.5 were more similar to each other and distinct from broth at pH 7.4 (Fig. S2), altogether suggesting that acidified broth might more closely resemble the intracellular environment of *P. aeruginosa*.

Regardless of the effect on rifampicin’s concentration-response and restoration of the synergy of the two-drug combination, the acidification of broth also brought about a similar increase in the efficacy of polymyxin B by one order of magnitude (Fig. 2A; Fig. S1B). In addition, the shared increase in efficacy upon acidification of broth was coupled to increased potency (lower *EC_50_*) of both drugs (Fig. 2B). The discrepancy between the favorable effect of acidic pH on polymyxin B activity in broth and polymyxin B’s low intracellular efficacy (Fig. 2B; Fig. S1B) prompted us to explore if pH contributes to the efficacy of intracellular killing of bacteria inside vacuoles. Indeed, increasing the vacuolar pH by chloroquine (33) decreased the efficacy of both polymyxin B and rifampicin (Fig. S1E and F). This suggests that the intracellular activity of polymyxin B is low despite the potentiating effect of acidic pH in the vacuole.

To establish if our findings apply more broadly to other strains of *P. aeruginosa*, we tested another reference strain (PAO1) and a clinical isolate (PA04/202; Fig. S3), rifampicin-resistant laboratory strains (Fig. S11; PA14 and isogenic mutants with a > 40-times higher intracellular *EC_50_* for rifampicin, Fig. 2B), and polymyxin B-resistant clinical isolates (Fig. S12; 80–120 times higher intracellular *EC_50_* for polymyxin B, Fig. 2B). Across all the strains, the results were similar to ATCC27853: (i) polymyxin B had higher efficacy in broth compared with intracellular environment, (ii) rifampicin’s efficacy was never higher in broth, and (iii) intracellular synergy of combining the two drugs was unequivocal. Most importantly, the synergy was not interrupted by resistance towards individual drugs (Fig. S11 and S12). Acidification enhanced synergy in all strains except PA14, where the effect was negligible. Overall, only the potentiating effect of pH on mono-treatments did not generalize from ATCC27853 to other *P. aeruginosa* strains.

### The combination is synergistic against a range of intracellular Gram-negative ESKAPE pathogens

Given the increased efficacy of the rifampicin-polymyxin B combination on intracellular *P. aeruginosa*, we asked if this generalizes to intracellular forms of other ESKAPE Gram-negative bacteria, namely *A. baumannii*, *K. pneumoniae*, and *E. coli*.

Across other ESKAPE pathogens, similarly to *P. aeruginosa*, although polymyxin B was more effective in broth compared with the intracellular environment, rifampicin’s efficacy in broth was less or similar to that in the intracellular context (Fig. 2A; Fig. S4 to S6). Acidification had a negligible effect on *E_max_* and, if anything, decreased the efficacy of both drugs.

Regarding single-drug potencies (Fig. 2B), acidification had no effect against *E. coli* and increased the potency of rifampicin only against *A. baumannii* (seven times). In contrast, low pH decreased the potency of polymyxin B against *K. pneumoniae* (12–30 times) and *A. baumannii* (6–7 times). Finally, rifampicin showed good activity against *A. baumannii*—compared with *P. aeruginosa* ATCC27853, rifampicin was some 8 times (*EC_50_* about 2 mg/L in intracellular and neutral broth) to 20 times (*EC_50_* 0.3 mg/L in acidic broth) more potent against both isolates of *A. baumannii* (Fig. S2B).

The drug combination was synergistic against all three species, and, excluding clinically irrelevant high concentrations, synergy was generally at or around the MICs of the two drugs. The effect of pH on synergies was concordant with *P. aeruginosa—*at pH 5.5, compared with pH 7.4, there were more synergistic locations across the tested concentration space (Fig. S7A). The only mild exceptions were *A. baumannii* A113 and *P. aeruginosa* PA04/202, for which the number of synergistic concentrations decreased slightly upon acidification.

**Fig 3:**
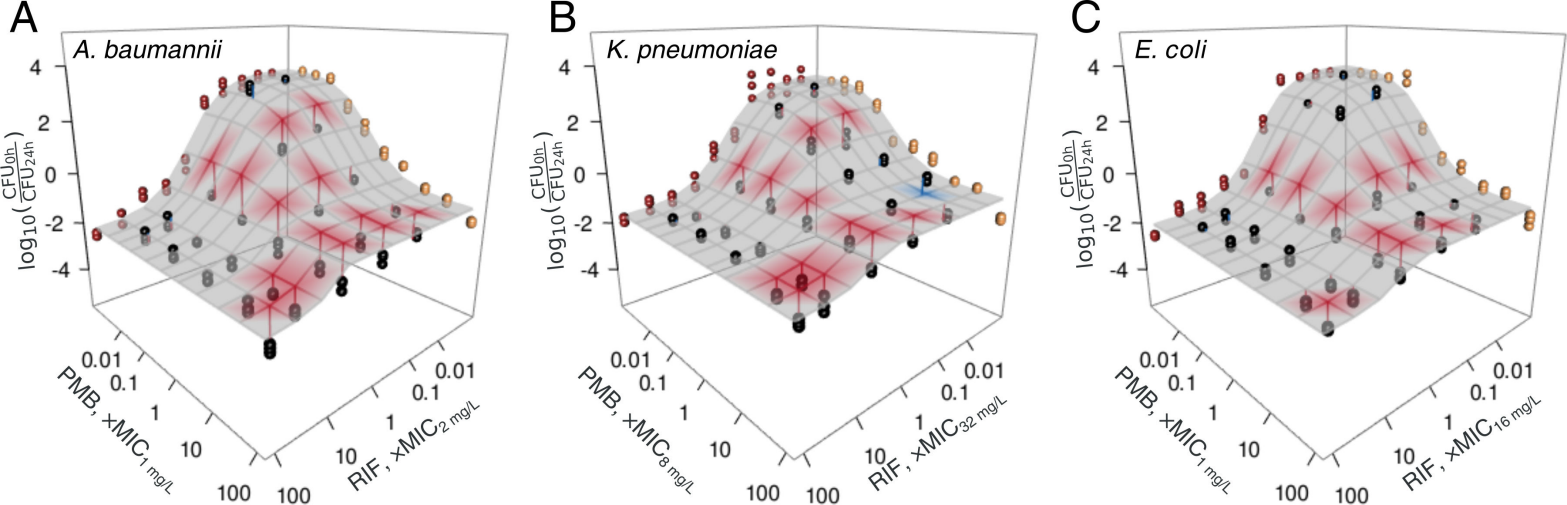
Rifampicin-polymyxin B combination is synergistic against other intracellular ESKAPE bacteria. Intracellular activity of polymyxin B, rifampicin, or their combination is shown against clinical isolates of (**A**) *A. baumannii*, (**B**) *K. pneumoniae*, and (**C**) *E. coli*. The experimentally measured outcome of the two-drug combination on intracellular bacteria is shown with black circles (each circle represents a biological replicate of experiments performed in three technical replicates). The red lines signify synergy, that is, connect points to the surface if the measured CFU are below the expectation surface. Conversely, blue indicates antagonism. The surface was colored if the difference from expectation was statistically significant (*P* < 0.05 from a bootstrapped approximation of the expected result under Loewe’s null).

Taken together, the rifampicin-polymyxin B combination is synergistic against intracellular clinical isolates of *A. baumannii*, *K. pneumoniae*, and *E. coli* (Fig. 3), with a relevant synergy region in concentration space at or around the drugs' MIC. Lowering pH increases the potency of rifampicin against *A. baumannii* and enhances synergy against all three species.

### Reverse genetics identifies mutants with altered sensitivity against rifampicin-polymyxin B combination

We turned to chemical genetics (34) to account for the synergy in molecular terms—potentially beyond a nonspecific increase in membrane permeability by polymyxin B. To date, an ordered transposon library is not available for ATCC27853 but has been established for *P. aeruginosa* strains PAO1 (35, 36) and PA14 (31). We chose PA14 because it forms less biofilm than PAO1 (37), which makes it a better strain for quantifying growth⁠ (the potential of combination to fight biofilm, an important clinical issue, is out of scope for the current study). In addition, PA14 is a highly virulent strain capable of infecting mammalian hosts and invertebrates, making it an attractive model for studying *P. aeruginosa* infection.

Although the PA14 strain had dramatically reduced sensitivity towards rifampicin, we decided to continue with the Tn-library because, apart from elevated MIC, insensitivity did not affect synergy (Fig. S11). In addition, preliminary tests on agar medium showed that the PA14 library strain’s growth—that is, the readout for chemical genetics—was inhibited by rifampicin at sub-MIC concentrations (levels close to the MIC of other *P. aeruginosa* strains). The primary and frequent mechanism for rifampicin resistance is point mutations in RNA polymerase (*rpoB* gene), each of which gradually reduces the binding affinity of rifampicin, causing the drug to dissociate from RNA polymerase (24, 38). Any fitness cost of such mutations is easily offset by compensatory mutations (39, 40), arguably minimizing any polar/secondary effects of the resistance.

**Fig 4:**
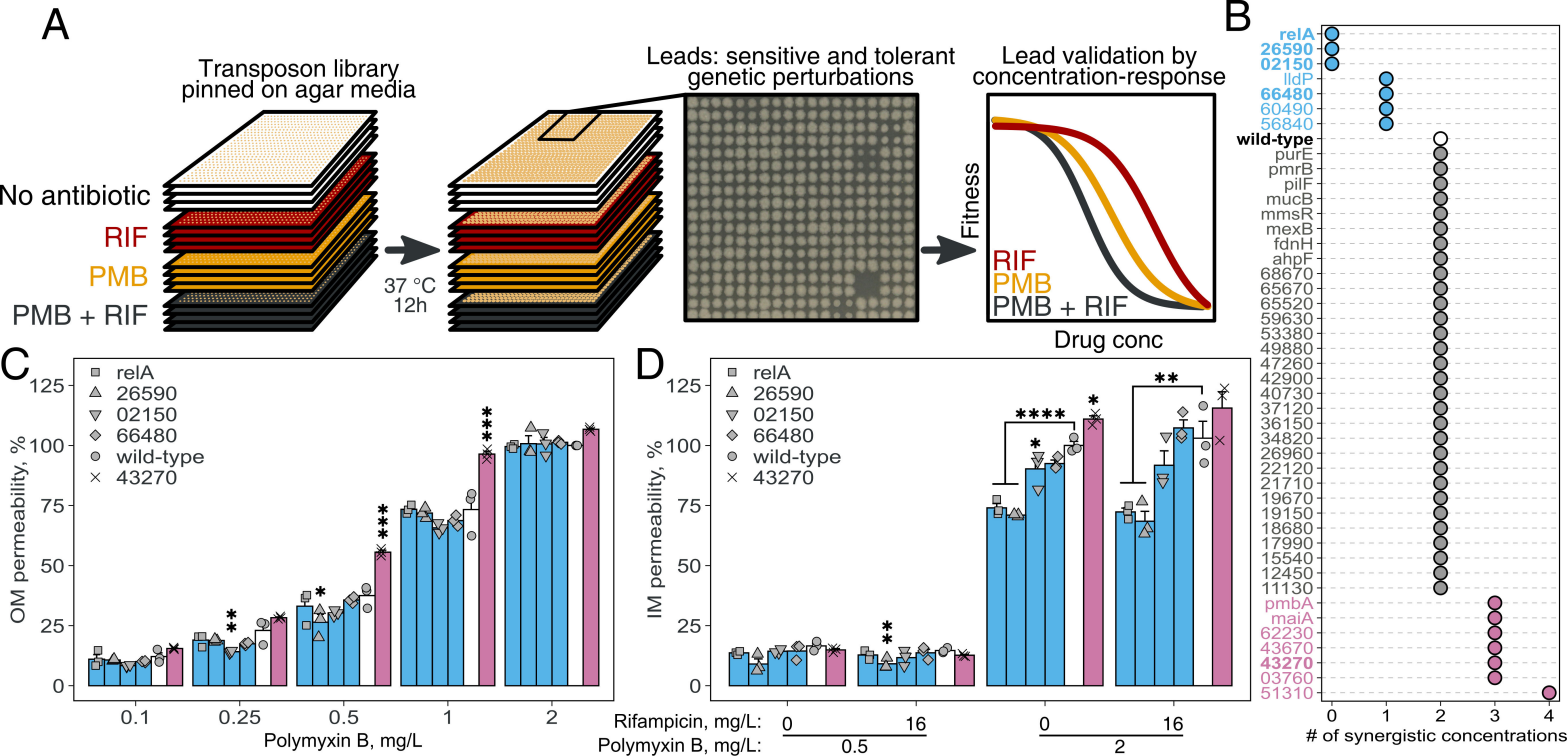
Reverse genetics identifies *P. aeruginosa* mutants with altered sensitivity towards rifampicin-polymyxin B combination. (**A**) *P. aeruginosa* PA14 ordered transposon library was pinned on an agar medium prepared with and without antibiotics. After incubation at 37°C, the plates were imaged, and growth was quantified using colony opacity. (**B**) Sensitivity to the combination was measured by counting how many of the eight tested concentration levels showed synergy. Increased (reddish purple), decreased (blue), and unchanged sensitivity (gray) are shown relative to the wild-type (white) with two synergistic concentration levels. (**C**) Outer membrane (OM) permeability upon increasing concentrations of polymyxin B. The color code is the same as in (**B**). (**D**) Inner membrane (IM) permeability upon different concentrations of polymyxin B alone and in combination with rifampicin. The color code is the same as in (**B**). In (**C**) and (**D**), error bars are SEM of 3 replicates from three independent experiments, and asterisks indicate *P*-values from a one-way ANOVA with Dunnett’s post hoc test comparing strains with wild-type (* <0.05, ** <0.01, *** <0.001, **** <0.0001).

We pinned the *P. aeruginosa* transposon library on agar media prepared with or without antibiotics; we used regular LB and LB buffers at pH 5.5 by 100 mM MES in parallel (for details, see Materials and Methods). We took pictures of the plates after incubation for 12 h at 37°C (Fig. 4A) and analyzed the images using Iris (41). Using colony opacity as a proxy for growth, we derived fitness—a relative growth in a given condition compared with the same mutant on a no-drug plate. Such a comparison of the mutant with itself allows us to estimate the treatment effect without interference from its proclivity for growth and the effects of position within a plate (42).

Combining results from neutral (Fig. S8A) and pH 5.5 (Fig. S8B) media, we found 57 mutants with altered sensitivity to the rifampicin-polymyxin B concentration. This entails 53 different genes. The difference between the numbers reflects the library composition—some mutants have the transposon landing site in other parts of the same gene (three of the 53 genes were hit by three, two, and two transposons, respectively). A discordant phenotype between same-gene mutants arises with a high frequency for multiple reasons, most notably due to the more or less dispensable nature of different regions of the same gene. However, if such mutants have a concordant phenotype, it increases the statistical power to call the gene a hit. In doing so, performing the analysis at the gene level, we found 17 additional genes inflicted. That said, we limited our analysis to the initial 53 genes (to work directly with the mutants from the library in the following experiments).

GO term enrichment analysis pointed to membrane processes—including the known polymyxin resistance element *pmrB* (43, 44)—and metabolism (Tables S1 and S2). Protein-protein interaction networks insinuated the same (Fig. S8C). In addition, *P. aeruginosa* harbors 12 resistance-nodulation-division (RND) type efflux systems, of which the best studied MexAB-OprM (5) is constitutively expressed and confers resistance to a wide range of structurally diverse antimicrobials (45). Accordingly, we identified that *mexB* had an increased sensitivity towards the combination.

Of the 53 genes, we next interrogated the 45 strongest affected mutants in liquid medium (LB Lennox) following growth by optical density at multiple drug concentrations. Briefly, the stationary phase culture was diluted 100-fold in LB (pH 7.4), distributed 30 µL/well at 384 wells per microtiter plate density, and growth was monitored by measuring OD_620_ every 30 min. We derived dose-response curves from areas under the log-transformed growth curve up to 15 h (Fig. S9) and calculated the drug-drug interaction score (as described in Materials and Methods).

Of the 45 transposon mutants closely examined in the liquid growth assay, 14 showed altered sensitivity to the drug combination (Fig. 4B; Table 1; Fig. S10). Seven had decreased synergy (four with less synergy, and three had lost all synergy), whereas the other seven had increased synergy. We excluded one mutant (transposon in *sahH*) from the analysis as it grew poorly in liquid broth. Nine of the genes had been characterized (*relA*, 26590, *lldP*, 60490, 03760, 43270, *maiA*, 43670, and *pmbA*), and five genes were hypothetical (02150, 66480, 56840, 51310, and 62230); for the latter, we provide the description based on conserved protein domains (Table 1). Four of the nine characterized had known *E. coli* orthologs (*relA*, *glcA*, *selU*, and *pmbA*) (Table 1).

**TABLE 1:** The validated subset of mutants had altered sensitivity toward the combination^*a*^

PA gene	EC ortholog	Synergy	Description
02150		None	Serine phosphatase RsbU, regulator of sigma subunit
relA	relA	None	(p)ppGpp synthetase
26590		None	GntR family transcriptional regulator
66480		Less	Predicted ATPase involved in chromosome partitioning
56840		Less	Predicted thiol oxidoreductase
IldP	glcA	Less	L-lactate permease
60490		Less	Cytochrome c
03760		More	Sodium:solute symporter
43270	SelU	More	tRNA 2-selenouridine synthase
51310		More	Predicted redox protein, regulator of disulfide-bound formation
maiA		More	Maleylacetoacetate isomerase
43670		More	Sensor/response regulator hybrid
pmbA	pmbA	More	PmbA protein
62230		More	Predicted kinase

^*a*^ Only four genes have *E. coli* orthologs. Five genes (02150, 66480, 56840, 51310, and 62230) encode hypothetical proteins for which we used conserved protein domains to predict the function.

### Identified mutants have altered inner and outer membranes

Having used two different growth-based assays to identify 53 mutants (by colony size at pH 7.4 and pH 5.5) and validate (by turbidity in liquid medium at pH 7.4) 14 mutants with altered sensitivity toward the combination, we next interrogated the drug-drug interaction in a concentration-resolved manner on the killing of bacteria (i) in intracellular infection experiment, (ii) in broth at pH 7.4, and (iii) in broth at pH 5.5. In addition, we further delineated some physiological properties of five mutants (*relA*, 26590, 02150, 66480, and 43270).

Although not vastly different from wild-type, mutants with a transposon in 43270 and 66480 had more and fewer regions of synergy, respectively (Fig. S11). The killing of mutants—during intracellular infection, in broth at pH 7.4, and in broth at pH 5.5—was thus concordant with our growth-based validation experiments (Fig. 4B; Fig. S9 and S10).

We next asked if mutants differ in their sensitivity toward the combination due to altered permeability of the outer membrane (OM), inner membrane (IM), or both, using nitrocefin and propidium iodide (PI) assay, respectively. Nitrocefin is a chromogenic substrate that cannot cross intact OM; however, when the OM is disrupted, nitrocefin reaches the periplasmic space, where periplasmic β-lactamases hydrolyze it, changing its color from yellow to red (46). PI is a dye that shows a 20-fold to 30-fold increase in fluorescence when it binds to DNA or RNA, but it cannot cross intact bacterial membranes (neither OM nor IM).

Regarding outer membrane permeability (Fig. 4C), there was no difference between mutants at the lowest (0.1 mg/L) and highest (2 mg/L) polymyxin B concentrations. However, the outer membrane of mutant 43270 was significantly more sensitive to polymyxin B at intermediate concentrations than that of the wild-type (or the rest of the mutants). Conversely, 02150 and 26590 were less sensitive than the wild-type. The relative sensitivity pattern was similar to that of polymyxin B nonapeptide (Fig. S13A), a 100-fold less bactericidal derivative of polymyxin B (47), suggesting that the effects we observed are independent of the killing activity of polymyxin B.

Regarding inner membrane permeability (Fig. 4D), for polymyxin B at 1× MIC—whether alone or combined with rifampicin—mutant 43270 was more sensitive. In contrast, *relA*, 26590, and 02150 were less sensitive. Transposon insertion in *relA* and 26590 reduced inner membrane permeabilization by 25%, protecting against polymyxin B and the rifampicin-polymyxin B combination.

In conclusion, when exposed to polymyxin B alone or combined with rifampicin, the isolated mutants show a positive correlation between the synergy and IM/OM permeabilization. However, these mutants were indistinguishable in terms of binding of polymyxin B to lipid A of the LPS (Fig. S13B), inner membrane depolarization (Fig. S13D), and, quite intriguingly, of rifampicin accumulation (Fig. S13E).

### Identified mutants more sensitive to the rifampicin-polymyxin B combination show higher ROS production and lipid peroxidation when exposed to the combination

**Fig 5:**
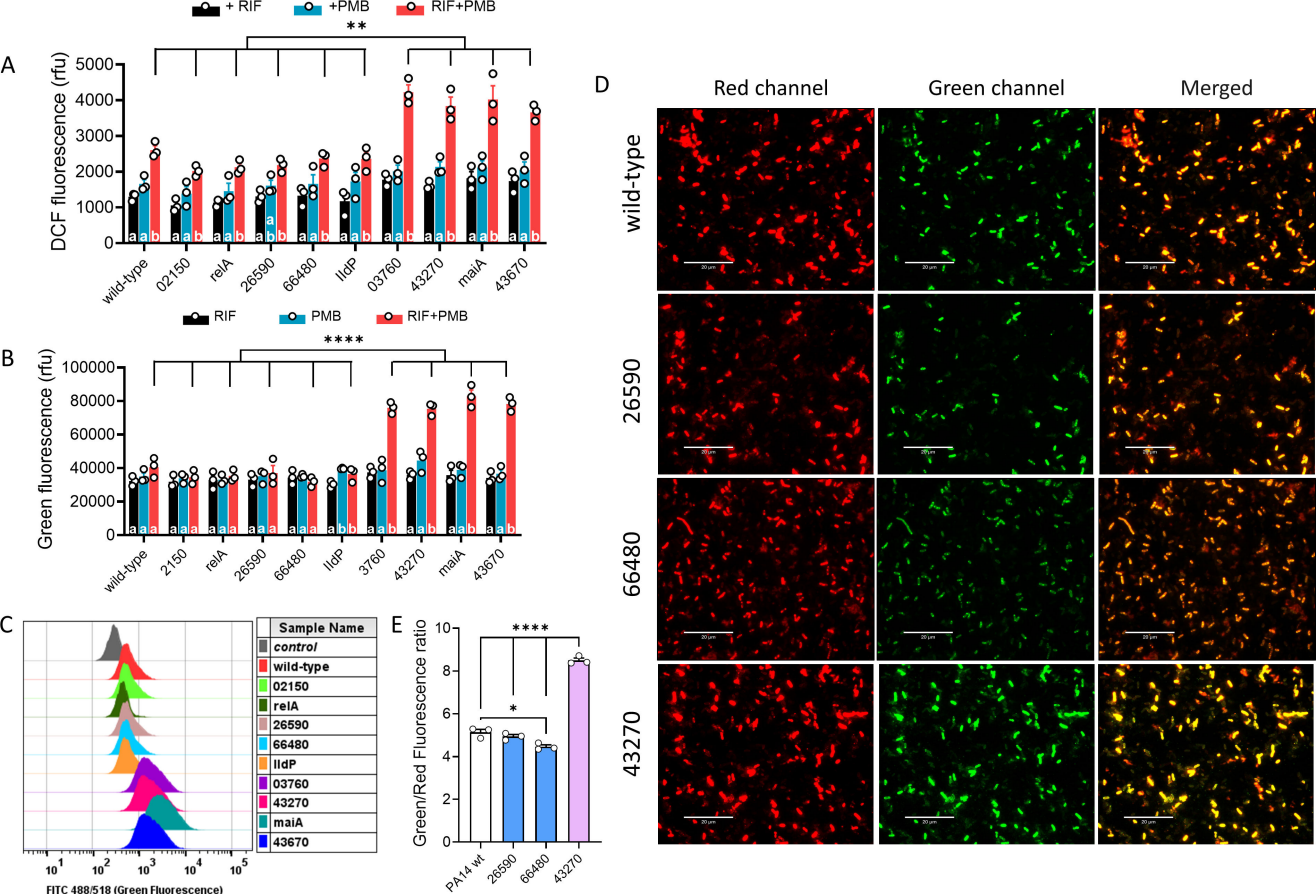
Rifampicin-polymyxin B combination therapy induces oxidative stress and membrane lipid peroxidation in *P. aeruginosa*. Bacteria were incubated with antibiotics at 1× MIC for 2 h. (**A**) ROS production was measured using a DCF fluorescence probe. (**B**) Lipid peroxidation was determined from the green fluorescence of the oxidation-sensitive probe C11-BODIPY^581/591^. (**C**) Flow cytometry analysis of green fluorescence intensity (FITC filter). Control: wild-type cells stained with C_11_-BODIPY^581/591^, without treatment. (**D**) Fluorescence microscopy of wild-type and specific mutants stained with C_11_-BODIPY^581/591^ and incubated with the combination of rifampicin and polymyxin B. Red fluorescence indicates non-oxidized lipids, and green fluorescence indicates oxidized lipids. The red-to-green shift was most pronounced in the 43270 mutant (more synergy in this mutant). Scale bars: 20 µm. (**E**) Fluorescence signal from microscopic images, quantified as the mean green/red fluorescence ratio. In (**A**), (**B**), and (**E**), error bars are SEM of 3 replicates from three independent experiments, and asterisks indicate *P*-values from a two-way ANOVA (**A and B**) or one-way ANOVA (**E**) with Tukey post hoc test comparing different strains exposed to the same treatment (*, *P* < 0.05, ** <0.01, **** <0.0001), whereas different letters highlight differences between treatments in each strain (*P* < 0.05).

Although many mutants with more regions of synergy have unidentified defects, we noticed that one of them, namely 43270, harbors mutations in tRNA 2-selenouridine synthase. Recent studies have highlighted the critical role of tRNA modifications in cellular stress responses. For example, tRNA 2-selenouridine synthase is crucial for maintaining translational accuracy and cellular stress adaptation by modifying wobble uridine, which enhances codon recognition and protein synthesis fidelity. Loss of this function can lead to increased mistranslation, resulting in protein misfolding and hypersensitivity to oxidative stress (48, 49). Accordingly, the analysis of GO terms highlights oxidative stress, with the top two terms related to it (Table S2). Lipids are one of the targets of oxidative damage (50). We therefore wondered whether synergy in the rifampicin-polymyxin B combination could occur through mechanisms involving oxidative stress and membrane lipid peroxidation. ROS levels were detected using the oxidation-sensitive probe DCF (51) and lipid peroxidation by following the shift from red to green fluorescence of C11-BODIPY^581/591^ (52). A significant increase in ROS levels was observed in all strains exposed to the combination of rifampicin and polymyxin B compared with those exposed to a single drug, but the difference was markedly higher in mutants with more regions of synergy (03760, 43270, maiA, and 43670) (Fig. 5A). Higher levels of lipid peroxidation were also measured in these same mutants following combined treatment with rifampicin and polymyxin B (Fig. 5B). This more marked shift of red to green fluorescence of C11-BODIPY^581/591^ indicative of lipid peroxidation was confirmed in flow cytometry (Fig. 5C) and fluorescence microscopy (Fig. 5D and E).

These results suggest that oxidative stress may play a key role in the enhanced bactericidal activity observed with the combination therapy.

## DISCUSSION

Combining polymyxins and rifampicin is one of the most discussed treatments against multidrug-resistant Gram-negative bacteria (53). We report here that the rifampicin and polymyxin B combination shows potent synergy against *P. aeruginosa*, *A. baumannii, E. coli*, and *K. pneumoniae* (Fig. 1B through D and 3; Figs. S3 to S6), including intracellular forms of bacteria and their clinical isolates. As reported previously for colistin (54, 55), synergy is retained even against the polymyxin B and rifampicin-resistant strains (Figs. S11 and S12).

We reveal that rifampicin (i) has good efficacy against *A. baumannii*, and (ii) contrary to other antibiotics, it is a drug with similar or higher potency and efficacy in an intracellular environment compared with a neutral laboratory medium. We show for *P. aeruginosa* that the medium at pH 5.5 might be a better approximation for the intracellular environment than the same medium at pH 7.4, both for the concentration-response relationship (Fig. S1A) and when following treatment effect in time (Fig. S2).

The effect of acidification, however, is more complex. Previous studies show that low pH increases the effectiveness of rifampicin against *Mycobacterium smegmatis* (56), and similar effects are seen here in *P. aeruginosa* and *A. baumannii* (Fig. 2B). However, acidification does not affect rifampicin potency against *E. coli* and actually reduces the potency of polymyxin B against *K. pneumoniae* and *A. baumannii* (Fig. 2B). In contrast, lowering the pH increases polymyxin B effectiveness in *P. aeruginosa*, that is, further amplifying the difference between the intracellular environment and broth. This makes an acidic medium, rather than a neutral one, less suitable specifically for studying polymyxin B intracellular pharmacodynamics.

Compared with neutral broth, synergy is potentiated by an acidic environment, that is, in an intracellular environment and an acidic broth (Fig. S7). Importantly, we observe that synergy increases the potency of the treatment, that is, around the MIC of the mono-treatments. In contrast, the synergistic gain in efficacy is of secondary importance as it happens at physiological unfeasibly high drug concentrations, if at all.

We identified a set of transposon mutants with altered sensitivity toward the combination. As polymyxin B targets membranes and rifampicin affects transcription, the identified mutants have an insertion in genes primarily involved in the two processes.

All the mutants without discernible synergy had transposon insertion in transcription regulators (Fig. 4B): (i) *relA* encodes a (p)ppGpp synthetase, a stringent response regulator that orchestrates bacterial transcription at the global level (57–59); (ii) 02150 is a regulator of sigma subunit of RNA polymerase, as suggested by a domain from the family of serine phosphatase RsbU (60); and (iii) 26590 encodes a transcription factor from the GntR family.

Fast-growing bacteria have a set of genes transcribed at a high rate, which RelA rapidly curtails upon perturbations (57–59), driving the accumulation of (p)ppGpp (61). Our current work suggests that perturbation of such capacity renders strains more sensitive to both rifampicin and polymyxin B (Fig. S9) as if removing an obstacle that synergy overcomes. This generic explanation lacks details for now, except that *relA* defective strains have altered inner membrane permeability (Fig. 4D). Curiously, a line of work connects the effect of rifampicin and the stringent response over the shared effect on replication (62, 63), and the accumulation of nucleotides and amino acids during the combination treatment (64, 65) suggests that cells are in a state of metabolic and/or growth arrest.

Although we could not observe a difference in rifampicin uptake for identified mutants, they had altered membrane properties (Fig. 4C and D) and showed higher ROS levels and lipid peroxidation when exposed to the rifampicin-polymyxin B combination (Fig. 5), which correlates with the susceptibility toward the combination (Fig. 4B). The potential role of oxidative stress was also supported by the GO term enrichment analysis of chemical-genetics experiments (Table S2). Of note, the oxidative stress induced by polymyxins and rifampicin may promote the oxidation of Fe^2+^ to Fe^3+^ via the Fenton reaction (66). This warrants further investigation, as ferroptosis has been implicated in acute kidney injury caused by polymyxins (67) and in the rifampicin-induced hepatotoxicity (68).

Some limitations of our work need to be acknowledged. Most of the genes associated with modified susceptibility to the drug combination in the transposon library code for proteins with unknown functions, preventing us from further investigating their potential link with the observed phenotype. Also, we did not extend our study to other polymyxins or rifamycins. Finally, we used a single model of phagocytic cells for intracellular infection and cannot exclude that the fate of bacteria may differ in other cell types, potentially influencing the extent of the synergies observed here.

Overall, our work indicates that rifampicin could be refactored as a potent agent against hard-to-treat Gram-negative bacteria if administered together with sub-MIC concentrations of polymyxin B, including against intracellular forms of infection. Importantly, our data suggest a link between membrane structure alterations and oxidative damage sensitivity, further emphasizing the therapeutic potential of leveraging such stress responses in combating multidrug-resistant infections. An exciting future direction could explore whether replacing polymyxins with other antimicrobial peptides (69, 70) leads to similar or even better synergistic effects.

## MATERIALS AND METHODS

### Reagents and media

Cation-adjusted Mueller Hinton, LB Lennox, and tryptic soy broth (TSB) were procured by Sigma. Solid media had 1.5% (wt/vol) agar (BD), except for chemical genetics, where agar was at 2% (wt/vol). The low pH version was obtained by acidification using HCl or by adding MES (Carl Roth) to pH 5.5 at 100 mM final. Rifampicin (Sigma; potency, 100%) stock was prepared in DMSO, and polymyxin B (Sigma; potency, 75%) and gentamicin (Sigma; potency, 65%) were prepared in water; subsequent dilutions were done in water or medium. In the case of experimental rifampicin concentrations higher than 1× MIC, the agar medium used for CFU counting was supplemented with 2 g/L charcoal powder to avoid the carry-over effect (Fig. S1C and D [71]). RPMI-1640 (Thermo) medium was used with or without 10% fetal calf serum (Sigma).

### Bacterial strains

*P. aeruginosa* reference strains ATCC27853, PAO1 (ATCC), and PA14 (36) were used. Clinical isolates, provided by Denis Pierard or Johan W. Mouton, included *P. aeruginosa* strains PA04/202, PA947, and PA1292; *K. pneumoniae* strains K74 and K58; *E. coli* strains E51 and E15; and *A. baumannii* strains A112 and A113.

### Intracellular infection

Intracellular infection of human THP-1 monocytic cells (ATCC) was performed as previously described (6), with cells used between the 5th and the 14th passages. Infected cells were then aliquoted 2 mL/well on 12-well plates with the appropriate antibiotics (alone or in combination) added to the wells at desired concentrations, incubated with antibiotics, and harvested as previously described (6). Bacterial abundance in CFU/mL was determined by colony counting after 24 h (and 48 h to capture late-appearing colonies). Total protein was determined from the samples to account for different yields in sample preparation (6). However, the variation in sample-to-sample yield was low. The data were fitted to a four-parameter sigmoidal equation with a Hill slope set at one in order to calculate pertinent pharmacodynamic parameters, namely *E_min_* (minimal efficacy, expressed in increase in bacterial counts from the initial post-phagocytosis inoculum, extrapolated for an infinitely low antibiotic concentration), *E_max_* (maximal efficacy expressed in decrease in bacterial counts from the initial post-phagocytosis inoculum, extrapolated for an infinitely large antibiotic concentration), *EC_50_* (concentration producing 50% of the maximal response), and *C_s_* (static concentration, that is, the concentration resulting in no apparent bacterial growth) (6).

The infection protocol for *P. aeruginosa* PA14 was modified to account for its gentamicin resistance (31), with amikacin (100 mg/L; 50 times the MIC) used instead to eliminate non-phagocytized bacteria.

### Reverse genetics

MHB-CA is a stringent medium for detecting membrane permeabilization by polymyxin B (69). We therefore opted for LB Lennox (5 g/L NaCl) or LB Lennox with 100 mM MES at pH 5.5. Plates were supplemented with 2% agar. Using an ordered PA14 transposon library (31), five independent copies of seeder plates were pinned in an array at 1536 density per rectangular Petri dish, incubated for 10 h at 37°C and then used to pin on the condition plates (two no-antibiotic controls; polymyxin B at 2 mg/L, i.e., 2× MIC; rifampicin at 16 mg/L, i.e., 1/128× MIC; polymyxin B at 2 mg/L + rifampicin at 16 mg/L) both at pH 7.4 and pH 5.5. After incubating plates for 12 h at 37°C, images were taken using spImager (S&P Robotics) and an 18-megapixel Canon EOS Rebel T3i camera. Colony characteristics were quantified with Iris software using colony opacity as a proxy for growth (41).

Data analysis consisted of the following five steps. First, quality control: (i) manual removal of swarming and low-quality plates, (ii) manual removal of colonies that were untransferred or missing due to problems with agar medium, (iii) programmatic removal of pairwise discordant (based on correlation test) plates after visual confirmation, and (ⅳ) programmatic removal of non-detected colonies, that is, colonies that were missing on only one of the replicates. Second, plate-to-plate correction: adjust the growth of colonies multiplicatively to bring every plate median to the global median. This step ensures that larger colonies on plates with better overall growth do not conflate with higher fitness values. Third, fitness calculation for each mutant. Growth was quantified using colony opacity (41). Fitness (*F*) was computed for each mutant as $F_{cond}\;=\frac{Opacity_{cond}}{Opacity_{NoAntibiotic}}\;;\;cond\;=\;\left\{rif,\;pmb,\;rif+pmb\right\}$. Note that this reasoning ensures that sick mutants with a growth defect are not mistaken for suffering the most from antibiotic treatment. Fourth, Bliss scores are found, which quantify the difference between measured and expected fitness for a combination. In the Bliss framework (72), probabilistic in principle, our estimated fitness is a complement of a drug effect, and expected combination fitness is the product of measured monotherapy fitnesses. The deviation from the model is quantified as a difference between the expected and measured: $Bliss\;score\;=\;F_{rif+pmb}-F_{rif}\times F_{pmb}$. Fifth, hypothesis testing of Bliss scores: (i) find a *P*-value using a one-sample *t*-test (i.e., what are the chances of finding the same or larger deviation from expected fitness (Bliss score = 0) if there is no interaction between the two drugs), and (ii) correct *P*-values for multiple testing (Benjamini-Hochberg correction).

### Validation of mutants

*P. aeruginosa* PA14 strains were grown in LB liquid and on LB agar medium. For mutant strains, 15 µg/mL gentamicin was added (gentamicin resistance is encoded by the transposon used to construct the library [31]). The day of the experiment, a colony was used to inoculate 3 mL of LB in a glass test tube. Fifteen mutants plus wild-type (isogenic to the mutant library) were tested simultaneously. Cultures were incubated for 7–8 h at 37°C, rolling at an angle for aeration. During that time, bacteria reach a stationary phase and stay there for a short period. Then, a 100× dilution of cultures was made in 2× LB, which was mixed 1:1 with drug conditions prepared in water. Cells were grown in an eight-step gradient of polymyxin B (from 6 to 0.25 µg/mL), rifampicin (from 288 to 12 µg/mL), and at the same drug concentrations combined element-wise (i.e., polymyxin B-rifampicin ratio of 1:48). Sixteen strains in 24 conditions resulted in one 384-well plate, 30 µL per well, covered with a transparent and breathable membrane. The microtiter plate was incubated in Synergy HTX (Biotek/Agilent) plate reader at 37°C, continuously shaken at the fastest speed setting, and OD_620nm_ was measured every 30 min for 14–16 h.

### Rifampicin accumulation

PA14 isogenic wild-type and mutants were inoculated in MHB-CA and grown overnight at 37°C at 130 rpm. Bacteria were harvested by centrifugation at 3,000 × *g*, and the pellet was resuspended in fresh MHB-CA. A fluorescent analog of rifampicin (73) was added at a final concentration of 20 mg/L. After 2 h of incubation at 37°C, 20 µL was removed for CFU counting; the rest was centrifuged at 3,000 × *g*, washed three times, and resuspended in cold PBS. All samples were then sonicated for 60 s and centrifuged at 20,000 × *g* for 7 min. The fluorescence in the supernatant was quantified at 470 nm (λ_ex_) and 522 nm (λ_em_).

### Membrane assays

Outer membrane permeability was determined by a nitrocefin assay (74). A single colony of *P. aeruginosa* from an overnight plate was grown in CA-MHB overnight, shaking at 130 rpm at 37°C. The culture was treated with 0.2 mg/L imipenem for 1 h to induce beta-lactamases. Bacteria were then collected by centrifugation and re-suspended in PBS at a final OD_620nm_ of 0.5; 100 µL of suspension was mixed with 50 µL nitrocefin (final concentration, 50 µg/mL) before the addition of 50 µL compounds of interest. Nitrocefin hydrolysis at 37°C was followed by measuring the absorbance at 490 nm for 1 h with an interval of 1 min using a SPECTRAmax M3 plate reader.

Inner membrane permeability was determined using the propidium iodide (PI) assay (75). Overnight bacterial cultures were centrifuged and resuspended in tris-phosphate buffer (110 mM NaCl; 7 mM KCl; 40 mM NH_4_Cl; 0.4 mM Na_2_HPO_4_; 62 mM Tris base; 0.2% glucose; pH adjusted to 7.5 with HCl) at an OD_620_ of 0.1. 5 mL of the bacterial suspension was mixed with 5 µL PI (final concentration, 6 µM) and incubated for 30 min in the dark, and 50 µL of this mixture was mixed with 50 µL of test compounds in a 96-well plate. After 60 min incubation, the fluorescence of PI was measured (λ_ex_/λ_em_: 535/617 nm) in a SpectraMax M3 plate reader.

The binding of polymyxin B to lipopolysaccharides (LPS) from *P. aeruginosa* was investigated using the BODIPY™-TR-cadaverine (BC) displacement assay, as previously described (76). BC (Thermo Fisher Scientific; final concentration, 5 µM) and bacteria (grown overnight at 130 rpm at 37°C in CA-MHB, pelleted and resuspended in PBS to OD_620_ 0.1) were mixed and kept for 30 min in the dark at room temperature, after which 50 µL of this mixture was incubated 30 min with 50 µL of test compounds in 96-well black plates. Then, fluorescence was read (λ_ex_/λ_em_: 580/620 nm) in a SpectraMax M3 plate reader.

### ROS measurements

ROS production in bacteria was measured using the oxidation-sensitive fluorescent probe 2′,7′-dichlorofluorescin diacetate (DCF) (51) (Sigma), using a protocol adapted from Johnson *et al*. (77). Overnight cultures were exposed to rifampicin or polymyxin B at their MIC alone or in combination for 2 h. The bacteria were then washed, resuspended in Hank’s balanced salt solution (Gibco), and incubated with 10  µM DCF for 30  min at 37°C. Fluorescence (λexc/λem: 485/530  nm) was recorded using a SpectraMax LS50 microplate reader and normalized to the OD_600 nm_ of the suspension.

### Membrane lipid peroxidation assay

Membrane lipid peroxidation was assessed using an oxidation-sensitive fluorophore, C11-BODIPY^581/591^ (Sigma), which shifts the emission maximum from 591 nm (red) to 510 nm (green) upon peroxidation (52). Overnight bacterial cultures were diluted into fresh LB medium and grown to mid-log phase (200 rpm, 37°C). Cells were then concentrated to 1 × 10^9^ CFU/mL and incubated with 2 µM C11-BODIPY^581/591^ for 2 h at room temperature to allow membrane incorporation of the probe. Then, 100 µL of the stained suspension was treated with rifampicin, polymyxin B (each at their MIC), or their combination for 2 h at 37°C in black 96-well microplates with transparent bottoms. Fluorescence was measured using a SpectraMax LS50 reader (red channel: excitation/emission = 530/590 nm; green channel: 470/510 nm), and values were normalized to the OD_620nm_ of the bacterial suspension.

For microscopy, samples were placed on 1% agarose pads and imaged using an ECHO Revolve microscope, with red fluorescence detected using λexc/λem 530–540/605–670 nm filters and green fluorescence, using λ_ex_/λ_em_ 470–40 nm and 525–550 nm filters.

For flow cytometry, 10 µL bacteria suspension was resuspended in 1 mL filtered PBS and analyzed using a FACSVerse cytometer (BD Biosciences). FITC (green; λexc/λem: 488/530 nm), and mCherry (red; λexc/λem: 561/610 nm) channels were used. To exclude doublets and damaged cells, we first plotted forward scatter width (FSC-W) against forward scatter area (FSC-A) and identified the main diagonal population corresponding to single cells. Events that deviated from this diagonal were gated out, as they typically represent cellular aggregates or exhibit abnormal pulse characteristics. We then applied a similar approach using side scatter width (SSC-W) versus side scatter area (SSC-A) to further refine the single-cell population and remove any remaining debris or abnormal events. mCherry-positive events were isolated, and data were analyzed using FlowJo 10.5.2 (TreeStar, Inc.) and reported as the green/red fluorescence ratio. The resulting flow cytometry profiles were displayed as the event frequency vs green fluorescence intensity. 

### Data analysis

Concentration-response curves were fit with a four-parameter log-logistic regression with a slope fixed to 1. Drug-drug interaction surface analysis, including associated statistical testing and 3D plotting, was carried out with the help of R package BIGL (78). Gene Ontology (GO) enrichment was calculated using GO terms from the Pseudomonas website (79) and Kolmogorov-Smirnov testing for statistical significance estimation. Protein-protein interaction (PPI) analysis was performed on data retrieved from the STRING database (80), and there were no data on PA14; hence, PAO1 data were used to build and analyze the network onto which then PA14 orthologs were mapped. *E. coli* orthologs were retrieved from OrthologDB (81). All intracellular infections and mechanistic studies (membrane effects, ROS, rifampicin accumulation) were performed in three replicates from three independent experiments and analyzed by ANOVA with appropriate post-hoc analysis using GraphPad software 9.4.1 (GraphPad Software Inc.). All data and analyses are available online at https://github.com/vvarik/pmb-rif.

## Data Availability

All data and analyzes are available online at https://github.com/vvarik/pmb-rif.

## SUPPLEMENTAL MATERIAL

Supplemental material aac.01319-25-s0001.pdf Tables S1 to S3; Fig. S1 to S13.

ASM does not own the copyrights to Supplemental Material that may be linked to, or accessed through, an article. The authors have granted ASM a non-exclusive, world-wide license to publish the Supplemental Material files. Please contact the corresponding author directly for reuse.

## References

[1] Tacconelli E, Magrini N, Carmeli Y, Harbarth S, Kahlmeter G, Kluytmans J, Mendelson M, Pulcini C, Singh N, Theuretzbacher U. 2017. Global priority list of antibiotic-resistant bacteria to guide research, discovery, and development of new antibiotics, p 318–327. Vol. 27. World Health Organization.

[2] De Oliveira DMP, Forde BM, Kidd TJ, Harris PNA, Schembri MA, Beatson SA, Paterson DL, Walker MJ. 2020. Antimicrobial resistance in ESKAPE pathogens. Clin Microbiol Rev 33:e00181–00119. doi:10.1128/CMR.00181-19 32404435 PMC7227449

[3] Kamaruzzaman NF, Kendall S, Good L. 2017. Targeting the hard to reach: challenges and novel strategies in the treatment of intracellular bacterial infections. Br J Pharmacol 174:2225–2236. doi:10.1111/bph.13664 27925153 PMC5481648

[4] Vincent J-L, Sakr Y, Singer M, Martin-Loeches I, Machado FR, Marshall JC, Finfer S, Pelosi P, Brazzi L, Aditianingsih D, Timsit J-F, Du B, Wittebole X, Máca J, Kannan S, Gorordo-Delsol LA, De Waele JJ, Mehta Y, Bonten MJM, Khanna AK, Kollef M, Human M, Angus DC, EPIC III Investigators. 2020. Prevalence and outcomes of infection among patients in intensive care units In 2017. JAMA 323:1478–1487. doi:10.1001/jama.2020.2717 32207816 PMC7093816

[5] Poole K. 2011. Pseudomonas aeruginosa: resistance to the max. Front Microbiol 2:65. doi:10.3389/fmicb.2011.00065 21747788 PMC3128976

[6] Buyck JM, Tulkens PM, Van Bambeke F. 2013. Pharmacodynamic evaluation of the intracellular activity of antibiotics towards Pseudomonas aeruginosa PAO1 in a model of THP-1 human monocytes. Antimicrob Agents Chemother 57:2310–2318. doi:10.1128/AAC.02609-12 23478951 PMC3632903

[7] Del Porto P, Cifani N, Guarnieri S, Di Domenico EG, Mariggiò MA, Spadaro F, Guglietta S, Anile M, Venuta F, Quattrucci S, Ascenzioni F. 2011. Dysfunctional CFTR alters the bactericidal activity of human macrophages against Pseudomonas aeruginosa. PLoS One 6:e19970. doi:10.1371/journal.pone.0019970 21625641 PMC3097223

[8] Chi E, Mehl T, Nunn D, Lory S. 1991. Interaction of Pseudomonas aeruginosa with A549 pneumocyte cells. Infect Immun 59:822–828. doi:10.1128/iai.59.3.822-828.1991 1671777 PMC258333

[9] Fleiszig SM, Zaidi TS, Fletcher EL, Preston MJ, Pier GB. 1994. Pseudomonas aeruginosa invades corneal epithelial cells during experimental infection. Infect Immun 62:3485–3493. doi:10.1128/iai.62.8.3485-3493.1994 8039920 PMC302982

[10] Garcia-Medina R, Dunne WM, Singh PK, Brody SL. 2005. Pseudomonas aeruginosa acquires biofilm-like properties within airway epithelial cells. Infect Immun 73:8298–8305. doi:10.1128/IAI.73.12.8298-8305.2005 16299327 PMC1307054

[11] Ha U, Jin S. 2001. Growth phase-dependent invasion of Pseudomonas aeruginosa and its survival within HeLa cells. Infect Immun 69:4398–4406. doi:10.1128/IAI.69.7.4398-4406.2001 11401979 PMC98512

[12] Schmiedl A, Kerber-Momot T, Munder A, Pabst R, Tschernig T. 2010. Bacterial distribution in lung parenchyma early after pulmonary infection with Pseudomonas aeruginosa. Cell Tissue Res 342:67–73. doi:10.1007/s00441-010-1036-y 20838814

[13] Malet K, Faure E, Adam D, Donner J, Liu L, Pilon SJ, Fraser R, Jorth P, Newman DK, Brochiero E, Rousseau S, Nguyen D. 2024. Intracellular Pseudomonas aeruginosa within the airway epithelium of cystic fibrosis lung tissues. Am J Respir Crit Care Med 209:1453–1462. doi:10.1164/rccm.202308-1451OC 38324627

[14] Baym M, Stone LK, Kishony R. 2016. Multidrug evolutionary strategies to reverse antibiotic resistance. Science 351:aad3292. doi:10.1126/science.aad3292 26722002 PMC5496981

[15] Brochado AR, Telzerow A, Bobonis J, Banzhaf M, Mateus A, Selkrig J, Huth E, Bassler S, Zamarreño Beas J, Zietek M, Ng N, Foerster S, Ezraty B, Py B, Barras F, Savitski MM, Bork P, Göttig S, Typas A. 2018. Species-specific activity of antibacterial drug combinations. Nature 559:259–263. doi:10.1038/s41586-018-0278-9 29973719 PMC6219701

[16] Co-ACTION. 2015. Developing combinations of CO-ACTIVE antimicrobials and non-antimicrobials - JPIAMR. Available from: https://www.jpiamr.eu/projects/co-action. Retrieved 28 Oct 2025.

[17] Wistrand-Yuen P, Olsson A, Skarp K-P, Friberg LE, Nielsen EI, Lagerbäck P, Tängdén T. 2020. Evaluation of polymyxin B in combination with 13 other antibiotics against carbapenemase-producing Klebsiella pneumoniae in time-lapse microscopy and time-kill experiments. Clin Microbiol Infect 26:1214–1221. doi:10.1016/j.cmi.2020.03.007 32224200

[18] Olsson A, Hong M, Al-Farsi H, Giske CG, Lagerbäck P, Tängdén T. 2021. Interactions of polymyxin B in combination with aztreonam, minocycline, meropenem, and rifampin against Escherichia coli producingNDM and OXA-48-group carbapenemases. Antimicrob Agents Chemother 65:e0106521. doi:10.1128/AAC.01065-21 34516251 PMC8597741

[19] Olsson A, Wistrand-Yuen P, Nielsen EI, Friberg LE, Sandegren L, Lagerbäck P, Tängdén T. 2020. Efficacy of antibiotic combinations against multidrug-resistant pseudomonas aeruginosa in automated time-lapse microscopy and static time-kill experiments. Antimicrob Agents Chemother 64:e02111–02119. doi:10.1128/AAC.02111-19 32179531 PMC7269485

[20] Bassetti M, Repetto E, Righi E, Boni S, Diverio M, Molinari MP, Mussap M, Artioli S, Ansaldi F, Durando P, Orengo G, Bobbio Pallavicini F, Viscoli C. 2008. Colistin and rifampicin in the treatment of multidrug-resistant Acinetobacter baumannii infections. J Antimicrob Chemother 61:417–420. doi:10.1093/jac/dkm509 18174197

[21] Motaouakkil S, Charra B, Hachimi A, Nejmi H, Benslama A, Elmdaghri N, Belabbes H, Benbachir M. 2006. Colistin and rifampicin in the treatment of nosocomial infections from multiresistant Acinetobacter baumannii. J Infect 53:274–278. doi:10.1016/j.jinf.2005.11.019 16442632

[22] Petrosillo N, Chinello P, Proietti MF, Cecchini L, Masala M, Franchi C, Venditti M, Esposito S, Nicastri E. 2005. Combined colistin and rifampicin therapy for carbapenem-resistant Acinetobacter baumannii infections: clinical outcome and adverse events. Clin Microbiol Infect 11:682–683. doi:10.1111/j.1469-0691.2005.01198.x 16008625

[23] Sterling TR, Njie G, Zenner D, Cohn DL, Reves R, Ahmed A, Menzies D, Horsburgh CR, Crane CM, Burgos M, LoBue P, Winston CA, Belknap R. 2020. Guidelines for the treatment of latent tuberculosis infection: recommendations from the national tuberculosis controllers association and CDC, 2020. MMWR Recomm Rep 69:1–11. doi:10.15585/mmwr.rr6901a1 PMC7041302 32053584

[24] Rothstein DM. 2016. Rifamycins, alone and in combination. Cold Spring Harb Perspect Med 6:a027011. doi:10.1101/cshperspect.a027011 27270559 PMC4930915

[25] Richter MF, Drown BS, Riley AP, Garcia A, Shirai T, Svec RL, Hergenrother PJ. 2017. Predictive compound accumulation rules yield a broad-spectrum antibiotic. Nature 545:299–304. doi:10.1038/nature22308 28489819 PMC5737020

[26] Vaara M. 1992. Agents that increase the permeability of the outer membrane. Microbiol Rev 56:395–411. doi:10.1128/mr.56.3.395-411.1992 1406489 PMC372877

[27] Poirel L, Jayol A, Nordmann P. 2017. Polymyxins: antibacterial activity, susceptibility testing, and resistance mechanisms encoded by plasmids or chromosomes. Clin Microbiol Rev 30:557–596. doi:10.1128/CMR.00064-16 28275006 PMC5355641

[28] Loewe S, Muischnek H. 1926. Über kombinationswirkungen.I mitteilung: hilfsmittel der fragestellung. Naunyn-Schmiedebergs Arch Exp Pathol Pharmakol 114:313–326. doi:10.1007/BF01952257

[29] Odds FC. 2003. Synergy, antagonism, and what the chequerboard puts between them. J Antimicrob Chemother 52:1. doi:10.1093/jac/dkg301 12805255

[30] Buyck JM, Lemaire S, Seral C, Anantharajah A, Peyrusson F, Tulkens PM, Van Bambeke F. 2016. In vitro models for the study of the intracellular activity of antibiotics. Methods Mol Biol 1333:147–157. doi:10.1007/978-1-4939-2854-5_13 26468107

[31] Liberati NT, Urbach JM, Miyata S, Lee DG, Drenkard E, Wu G, Villanueva J, Wei T, Ausubel FM. 2006. An ordered, nonredundant library of Pseudomonas aeruginosa strain PA14 transposon insertion mutants. Proc Natl Acad Sci U S A 103:2833–2838. doi:10.1073/pnas.0511100103 16477005 PMC1413827

[32] Pinhal S, Ropers D, Geiselmann J, de Jong H. 2019. Acetate metabolism and the inhibition of bacterial growth by acetate. J Bacteriol 201:e00147–00119. doi:10.1128/JB.00147-19 30988035 PMC6560135

[33] Poole B, Ohkuma S. 1981. Effect of weak bases on the intralysosomal pH in mouse peritoneal macrophages. J Cell Biol 90:665–669. doi:10.1083/jcb.90.3.665 6169733 PMC2111912

[34] Brochado AR, Typas A. 2013. High-throughput approaches to understanding gene function and mapping network architecture in bacteria. Curr Opin Microbiol 16:199–206. doi:10.1016/j.mib.2013.01.008 23403119

[35] Held K, Ramage E, Jacobs M, Gallagher L, Manoil C. 2012. Sequence-verified two-allele transposon mutant library for Pseudomonas aeruginosa PAO1. J Bacteriol 194:6387–6389. doi:10.1128/JB.01479-12 22984262 PMC3497512

[36] Jacobs MA, Alwood A, Thaipisuttikul I, Spencer D, Haugen E, Ernst S, Will O, Kaul R, Raymond C, Levy R, Chun-Rong L, Guenthner D, Bovee D, Olson MV, Manoil C. 2003. Comprehensive transposon mutant library of Pseudomonas aeruginosa. Proc Natl Acad Sci U S A 100:14339–14344. 14617778 10.1073/pnas.2036282100 PMC283593

[37] Mikkelsen H, McMullan R, Filloux A. 2011. The Pseudomonas aeruginosa reference strain PA14 displays increased virulence due to a mutation in ladS. PLoS One 6:e29113. doi:10.1371/journal.pone.0029113 22216178 PMC3245244

[38] Zhang Q, An X, Liu H, Wang S, Xiao T, Liu H. 2019. Uncovering the resistance mechanism of Mycobacterium tuberculosis to rifampicin due to RNA polymerase H451D/Y/R mutations from computational perspective. Front Chem 7:819. doi:10.3389/fchem.2019.00819 31850310 PMC6902089

[39] Hall AR, MacLean RC. 2011. Epistasis buffers the fitness effects of rifampicin- resistance mutations in Pseudomonas aeruginosa. Evolution 65:2370–2379. doi:10.1111/j.1558-5646.2011.01302.x 21790582

[40] Hughes D, Brandis G. 2013. Rifampicin resistance: fitness costs and the significance of compensatory evolution. Antibiotics (Basel) 2:206–216. doi:10.3390/antibiotics2020206 27029299 PMC4790335

[41] Kritikos G, Banzhaf M, Herrera-Dominguez L, Koumoutsi A, Wartel M, Zietek M, Typas A. 2017. A tool named Iris for versatile high-throughput phenotyping in microorganisms. Nat Microbiol 2:17014. doi:10.1038/nmicrobiol.2017.14 28211844 PMC5464397

[42] Auld DSP, Coassin PB, Coussens NPP, Hensley P, Klumpp-Thomas C, Michael S, Sittampalam GSP, Trask OB, Wagner BKP, Weidner JRP, Wildey MJP, Dahlin Jl Md P, et al.. 2004. Microplate selection and recommended practices in high-throughput screening and quantitative biology. In Markossian S, Grossman A, Arkin M, Auld D, Austin C, Baell J, Brimacombe K, Chung TDY, Coussens NP, Dahlin JL (ed),. Assay Guidance Manual, Bethesda (MD). 32520474

[43] McPhee JB, Lewenza S, Hancock REW. 2003. Cationic antimicrobial peptides activate a two-component regulatory system, PmrA-PmrB, that regulates resistance to polymyxin B and cationic antimicrobial peptides in Pseudomonas aeruginosa. Mol Microbiol 50:205–217. doi:10.1046/j.1365-2958.2003.03673.x 14507375

[44] Moskowitz SM, Brannon MK, Dasgupta N, Pier M, Sgambati N, Miller AK, Selgrade SE, Miller SI, Denton M, Conway SP, Johansen HK, Høiby N. 2012. PmrB mutations promote polymyxin resistance of Pseudomonas aeruginosa isolated from colistin-treated cystic fibrosis patients. Antimicrob Agents Chemother 56:1019–1030. doi:10.1128/AAC.05829-11 22106224 PMC3264203

[45] Van Bambeke F, Pagès J-M, Lee VJ. 2006. Inhibitors of bacterial efflux pumps as adjuvants in antibiotic treatments and diagnostic tools for detection of resistance by efflux. Recent Pat Antiinfect Drug Discov 1:157–175. doi:10.2174/157489106777452692 18221142

[46] Angus BL, Carey AM, Caron DA, Kropinski AM, Hancock RE. 1982. Outer membrane permeability in Pseudomonas aeruginosa: comparison of a wild-type with an antibiotic-supersusceptible mutant. Antimicrob Agents Chemother 21:299–309. doi:10.1128/AAC.21.2.299 6803666 PMC181877

[47] Vaara M, Vaara T. 1983. Polycations sensitize enteric bacteria to antibiotics. Antimicrob Agents Chemother 24:107–113. doi:10.1128/AAC.24.1.107 6414364 PMC185112

[48] Mitchener MM, Begley TJ, Dedon PC. 2023. Molecular coping mechanisms: reprogramming trnas to regulate codon-biased translation of stress response proteins. Acc Chem Res 56:3504–3514. doi:10.1021/acs.accounts.3c00572 37992267 PMC10702489

[49] Chan CTY, Pang YLJ, Deng W, Babu IR, Dyavaiah M, Begley TJ, Dedon PC. 2012. Reprogramming of tRNA modifications controls the oxidative stress response by codon-biased translation of proteins. Nat Commun 3:937. doi:10.1038/ncomms1938 22760636 PMC3535174

[50] da Cruz Nizer WS, Inkovskiy V, Versey Z, Strempel N, Cassol E, Overhage J. 2021. Oxidative stress response in Pseudomonas aeruginosa. Pathogens 10:1187. doi:10.3390/pathogens10091187 34578219 PMC8466533

[51] Wang H, Joseph JA. 1999. Quantifying cellular oxidative stress by dichlorofluorescein assay using microplate reader. Free Radic Biol Med 27:612–616. doi:10.1016/s0891-5849(99)00107-0 10490282

[52] Yoshida Y, Shimakawa S, Itoh N, Niki E. 2003. Action of DCFH and BODIPY as a probe for radical oxidation in hydrophilic and lipophilic domain. Free Radic Res 37:861–872. doi:10.1080/1071576031000148736 14567446

[53] Molina J, Cordero E, Pachón J. 2009. New information about the polymyxin/colistin class of antibiotics. Expert Opin Pharmacother 10:2811–2828. doi:10.1517/14656560903334185 19929704

[54] Armengol E, Kragh KN, Tolker-Nielsen T, Sierra JM, Higazy D, Ciofu O, Viñas M, Høiby N. 2023. Colistin enhances rifampicin’s antimicrobial action in colistin-resistant Pseudomonas aeruginosa biofilms. Antimicrob Agents Chemother 67. doi:10.1128/aac.01641-22 PMC10112245 36856424

[55] MacNair CR, Stokes JM, Carfrae LA, Fiebig-Comyn AA, Coombes BK, Mulvey MR, Brown ED. 2018. Overcoming mcr-1 mediated colistin resistance with colistin in combination with other antibiotics. Nat Commun 9:458. doi:10.1038/s41467-018-02875-z 29386620 PMC5792607

[56] Bartek IL, Reichlen MJ, Honaker RW, Leistikow RL, Clambey ET, Scobey MS, Hinds AB, Born SE, Covey CR, Schurr MJ, Lenaerts AJ, Voskuil MI. 2016. Antibiotic bactericidal activity is countered by maintaining pH homeostasis in Mycobacterium smegmatis. mSphere 1:e00176–00116. doi:10.1128/mSphere.00176-16 27579369 PMC4999920

[57] Gourse RL, Chen AY, Gopalkrishnan S, Sanchez-Vazquez P, Myers A, Ross W. 2018. Transcriptional responses to ppGpp and DksA. Annu Rev Microbiol 72:163–184. doi:10.1146/annurev-micro-090817-062444 30200857 PMC6586590

[58] Hauryliuk V, Atkinson GC, Murakami KS, Tenson T, Gerdes K. 2015. Recent functional insights into the role of (p)ppGpp in bacterial physiology. Nat Rev Microbiol 13:298–309. doi:10.1038/nrmicro3448 25853779 PMC4659695

[59] Hobbs JK, Boraston AB. 2019. (p)ppGpp and the stringent response: an emerging threat to antibiotic therapy. ACS Infect Dis 5:1505–1517. doi:10.1021/acsinfecdis.9b00204 31287287

[60] Voelker U, Dufour A, Haldenwang WG. 1995. The Bacillus subtilis rsbU gene product is necessary for RsbX-dependent regulation of sigma B. J Bacteriol 177:114–122. doi:10.1128/jb.177.1.114-122.1995 8002609 PMC176563

[61] Varik V, Oliveira SRA, Hauryliuk V, Tenson T. 2017. HPLC-based quantification of bacterial housekeeping nucleotides and alarmone messengers ppGpp and pppGpp. Sci Rep 7:11022. doi:10.1038/s41598-017-10988-6 28887466 PMC5591245

[62] Flåtten I, Skarstad K. 2009. DnaA protein interacts with RNA polymerase and partially protects it from the effect of rifampicin. Mol Microbiol 71:1018–1030. doi:10.1111/j.1365-2958.2008.06585.x 19170875

[63] Riber L, Løbner-Olesen A. 2020. Inhibition of Escherichia coli chromosome replication by rifampicin treatment or during the stringent response is overcome by de novo DnaA protein synthesis. Mol Microbiol 114:906–919. doi:10.1111/mmi.14531 32458540 PMC7818497

[64] Mahamad Maifiah MH, Zhu Y, Tsuji BT, Creek DJ, Velkov T, Li J. 2022. Integrated metabolomic and transcriptomic analyses of the synergistic effect of polymyxin-rifampicin combination against Pseudomonas aeruginosa. J Biomed Sci 29:89. doi:10.1186/s12929-022-00874-3 36310165 PMC9618192

[65] Zhao J, Han ML, Zhu Y, Lin YW, Wang YW, Lu J, Hu Y, Tony Zhou Q, Velkov T, Li J. 2021. Comparative metabolomics reveals key pathways associated with the synergistic activity of polymyxin B and rifampicin combination against multidrug-resistant Acinetobacter baumannii. Biochem Pharmacol 184:114400. doi:10.1016/j.bcp.2020.114400 33387481 PMC7906441

[66] Kohanski MA, Dwyer DJ, Hayete B, Lawrence CA, Collins JJ. 2007. A common mechanism of cellular death induced by bactericidal antibiotics. Cell 130:797–810. doi:10.1016/j.cell.2007.06.049 17803904

[67] Li H, Wang B, Wu S, Dong S, Jiang G, Huang Y, Tong X, Yu M. 2023. Ferroptosis is involved in polymyxin B-induced acute kidney injury via activation of p53. Chem Biol Interact 378:110479. doi:10.1016/j.cbi.2023.110479 37088170

[68] Zhou J, Tan Y, Hu L, Fu J, Li D, Chen J, Long Y. 2022. Inhibition of HSPA8 by rifampicin contributes to ferroptosis via enhancing autophagy. Liver Int 42:2889–2899. doi:10.1111/liv.15459 36254713

[69] Sánchez-Gómez S, Lamata M, Leiva J, Blondelle SE, Jerala R, Andrä J, Brandenburg K, Lohner K, Moriyón I, Martínez-de-Tejada G. 2008. Comparative analysis of selected methods for the assessment of antimicrobial and membrane-permeabilizing activity: a case study for lactoferricin derived peptides. BMC Microbiol 8:196. doi:10.1186/1471-2180-8-196 19014450 PMC2615442

[70] Ovchinnikov KV, Kranjec C, Telke A, Kjos M, Thorstensen T, Scherer S, Carlsen H, Diep DB. 2021. A strong synergy between the thiopeptide bacteriocin micrococcin P1 and rifampicin against MRSA in a murine skin infection model. Front Immunol 12:676534. doi:10.3389/fimmu.2021.676534 34276663 PMC8284338

[71] Wang G, Brunel J-M, Bolla J-M, Van Bambeke F. 2021. The polyaminoisoprenyl potentiator NV716 revives old disused antibiotics against intracellular forms of infection by Pseudomonas aeruginosa. Antimicrob Agents Chemother 65:e02028–02020. doi:10.1128/AAC.02028-20 33318000 PMC8092510

[72] Bliss CI. 1939. The toxicity of poisons applied jointly. Annals Applied Biol 26:585–615. doi:10.1111/j.1744-7348.1939.tb06990.x

[73] Wang G, Brunel J-M, Preusse M, Mozaheb N, Willger SD, Larrouy-Maumus G, Baatsen P, Häussler S, Bolla J-M, Van Bambeke F. 2022. The membrane-active polyaminoisoprenyl compound NV716 re-sensitizes Pseudomonas aeruginosa to antibiotics and reduces bacterial virulence. Commun Biol 5:871. doi:10.1038/s42003-022-03836-5 36008485 PMC9411590

[74] Borselli D, Lieutaud A, Thefenne H, Garnotel E, Pagès J-M, Brunel JM, Bolla J-M. 2016 Polyamino-isoprenic derivatives block intrinsic resistance of p. Aeruginosa to doxycycline and chloramphenicol in vitro. PLoS One 11:e0154490. doi:10.1371/journal.pone.0154490 27152508 PMC4859512

[75] Sautrey G, El Khoury M, Dos Santos AG, Zimmermann L, Deleu M, Lins L, Décout J-L, Mingeot-Leclercq M-P. 2016. Negatively charged lipids as a potential target for new amphiphilic aminoglycoside antibiotics: a biophysical study. J Biol Chem 291:13864–13874. doi:10.1074/jbc.M115.665364 27189936 PMC4919468

[76] Swain J, El Khoury M, Flament A, Dezanet C, Briée F, Van Der Smissen P, Décout J-L, Mingeot-Leclercq M-P. 2019. Antimicrobial activity of amphiphilic neamine derivatives: understanding the mechanism of action on Gram-positive bacteria. Biochim Biophys Acta Biomembr 1861:182998. doi:10.1016/j.bbamem.2019.05.020 31153908

[77] Johnson L, Mulcahy H, Kanevets U, Shi Y, Lewenza S. 2012. Surface-localized spermidine protects the Pseudomonas aeruginosa outer membrane from antibiotic treatment and oxidative stress. J Bacteriol 194:813–826. doi:10.1128/JB.05230-11 22155771 PMC3272965

[78] Van der Borght K, Tourny A, Bagdziunas R, Thas O, Nazarov M, Turner H, Verbist B, Ceulemans H. 2017. BIGL: Biochemically Intuitive Generalized Loewe null model for prediction of the expected combined effect compatible with partial agonism and antagonism. Sci Rep 7:17935. doi:10.1038/s41598-017-18068-5 29263342 PMC5738392

[79] Winsor GL, Griffiths EJ, Lo R, Dhillon BK, Shay JA, Brinkman FSL. 2016. Enhanced annotations and features for comparing thousands of Pseudomonas genomes in the Pseudomonas genome database. Nucleic Acids Res 44:D646–53. doi:10.1093/nar/gkv1227 26578582 PMC4702867

[80] Szklarczyk D, Gable AL, Nastou KC, Lyon D, Kirsch R, Pyysalo S, Doncheva NT, Legeay M, Fang T, Bork P, Jensen LJ, von Mering C. 2021. The STRING database in 2021: customizable protein-protein networks, and functional characterization of user-uploaded gene/measurement sets. Nucleic Acids Res 49:D605–D612. doi:10.1093/nar/gkaa1074 33237311 PMC7779004

[81] Whiteside MD, Winsor GL, Laird MR, Brinkman FSL. 2013. OrtholugeDB: a bacterial and archaeal orthology resource for improved comparative genomic analysis. Nucleic Acids Res 41:D366–76. doi:10.1093/nar/gks1241 23203876 PMC3531125

