# Supplementary material for: Synergistic activity of rifampicin and polymyxin B against intracellular Gram-negative ESKAPE pathogens involves bacterial membrane alterations and enhanced oxidative damages
Source: Antimicrob Agents Chemother. 2025 Nov 28;70(1):e01319-25. doi: 10.1128/aac.01319-25 (PMC12777556; doi:10.1128/aac.01319-25)
Supplement: Supplemental material — Tables S1 to S3; Fig. S1 to S13. [file aac.01319-25-s0001.pdf]

Title: **Synergistic activity of rifampicin and polymyxin B against intracellular Gram-negative ESKAPE pathogens involves bacterial membrane alterations and enhanced oxidative damages**

Authors: Vallo Varik[1,2,§*], Gang Wang[1*], George Kritikos[2,@], Manuel Banzhaf[3,£], Emilien Drouot[1,$], Alexandra Koumoutsi[2], Françoise Van Bambeke[1#]

[1] Pharmacologie cellulaire et moléculaire, Louvain Drug Research Institute, Université catholique de Louvain, Brussels, Belgium
[2] European Molecular Biology Laboratory, Heidelberg, Germany
[3] Newcastle University Biosciences Institute, Faculty of Medical Sciences, Newcastle University, Newcastle upon Tyne, UK

[§] present affiliation: Estonian Biofoundry, Institute of Bioengineering, University of Tartu, Tartu, Estonia
[@] present affiliation: European Food Safety Authority, Parma, Italy
[$] present affiliation: *Union chimique belge,* Brussels, Belgium

* Both authors contributed equally to this study.

Supplementary materials

Supplementary Table 1.

Screen hits' GO term enrichment for cell components.

GO ID	Term	P value
0016021	integral component of membrane	0.015
0005694	chromosome	0.072
0008076	voltage-gated potassium channel complex	0.140
0055052	ATP-binding cassette (ABC) transporter complex, substrate-binding subunit-containing	0.146
0009289	pilus	0.163
0033573	high-affinity iron permease complex	0.166
0005839	proteasome core complex	0.183
0005960	glycine cleavage complex	0.227
0030257	type III protein secretion system complex	0.252
0005615	extracellular space	0.253

Supplementary Table 2.

Screen hits' GO term enrichment for biological processes.

GO ID	Term	P value
0009236	cobalamin biosynthetic process	0.017
0055114	oxidation-reduction process	0.019
0009116	nucleoside metabolic process	0.024
0019700	organic phosphonate catabolic process	0.039
0017000	antibiotic biosynthetic process	0.040
0070475	rRNA base methylation	0.050
0009306	protein secretion	0.056
0044010	single-species biofilm formation	0.063
0019354	siroheme biosynthetic process	0.067
0044262	cellular carbohydrate metabolic process	0.074

Supplementary Figure 1

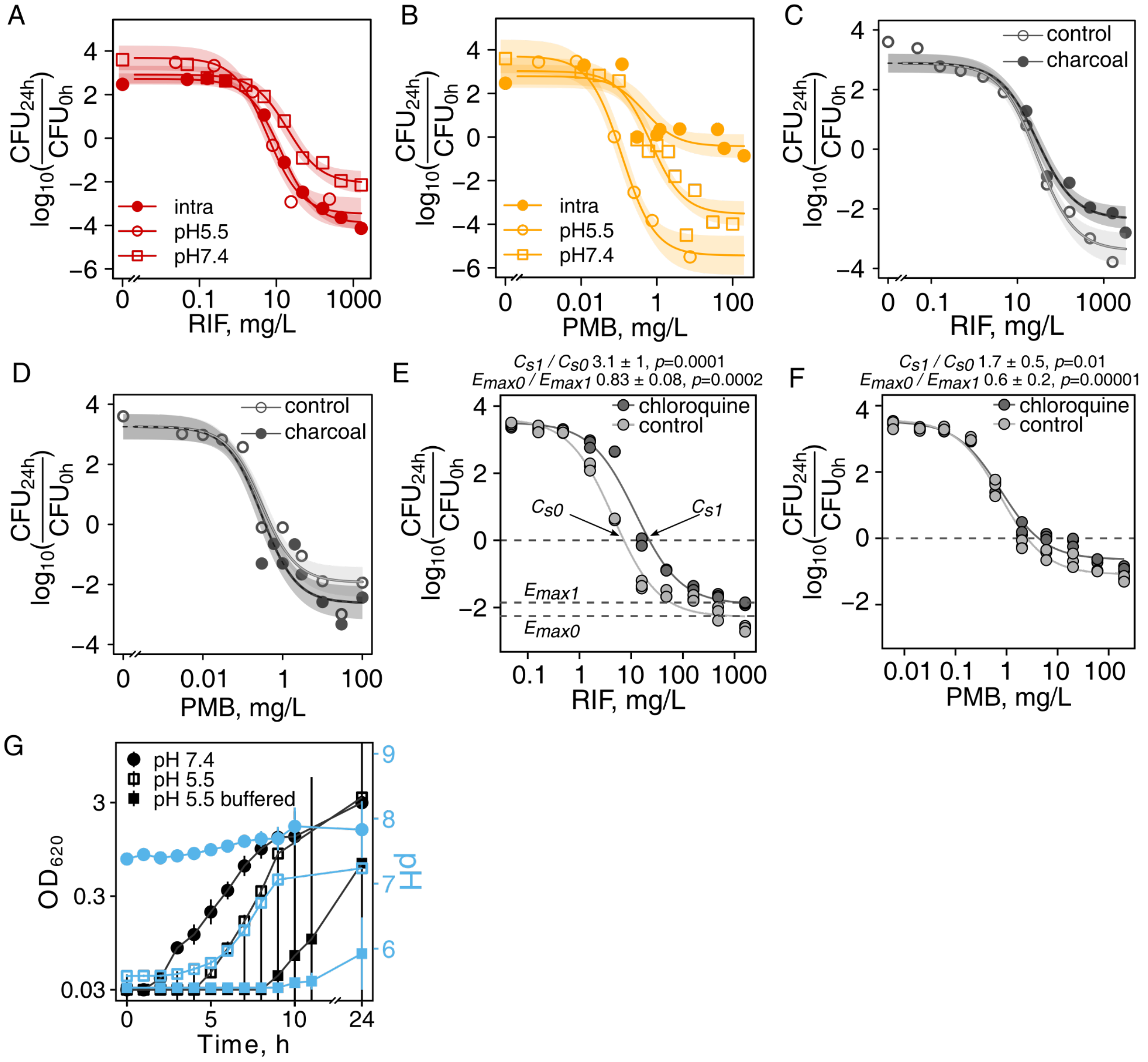

***P. aeruginosa* dose–response of monotherapies, role of charcoal and pH. (A)** *P. aeruginosa* ATC278553 was exposed to a range of rifampicin concentrations for 24 h, and CFU were counted after serial dilution of the samples. Points correspond to the arithmetic mean; lines correspond to a fit, a four-parametric logistic regression with a slope constrained to one; the shaded area is a 95% confidence interval for the fit. Intra: THP-1 monocytes infected with *P. aeruginosa*. pH 7.4: cation-adjusted Mueller Hinton. pH 5.5: cation-adjusted Mueller Hinton with 100 mM MES at pH 5.5. For experimental details, see Materials and methods. **(B)** Same as (A) but for polymyxin B. **(C-D)** Same as (A) and (B) in broth at pH 7.4, except at higher drug concentrations, the results are also shown for measuring CFUs on plates without 2 g/L charcoal (mean values are shown for n 4-7; shaded area is a 95% CI for the fit). **(E)** Rifampicin's concentration-dependent effect on intracellular *P. aeruginosa* ATCC27853 with and without chloroquine treatment to decrease the vacuolar pH. Statistically significant change +/- 95% CI is indicated above the plots for C_s (static concentration, i.e. concentration of no net increase in bacterial numbers) and E_{max} (maximal efficacy). **(F)** Same as (E) but for polymyxin B. **(G)** An *P. aeruginosa* overnight culture—grown in cation adjusted Mueller Hinton pH 7.4—was diluted to ~10^6 CFU/mL (OD_{620}=0.001) in the same broth 1) without any adjustment (pH 7.4), 2) pH brought to pH 5.5 by 1N HCl (pH 5.5), and 3) pH buffered at pH 5.5 by 100 mM MES (pH 5.5 buffered). Next, it was incubated at 37°C, 130 rpm, and measured turbidity and the pH of the supernatant at the time points indicated. pH values are plotted in blue on the right axis and CFU in black on the left axis. Points are arithmetic means (4-7 biological replicates on A and B; 3-5 biological replicates on C; 4 (pH 5.5 and pH 5.5 buffered) or 10 biological replicates (pH 7.4)), and error bars are 95% CI. P-values are from a two-sided t-test using heteroscedasticity consistent sandwich covariance estimators of a parametric regression.

Supplementary Figure 2

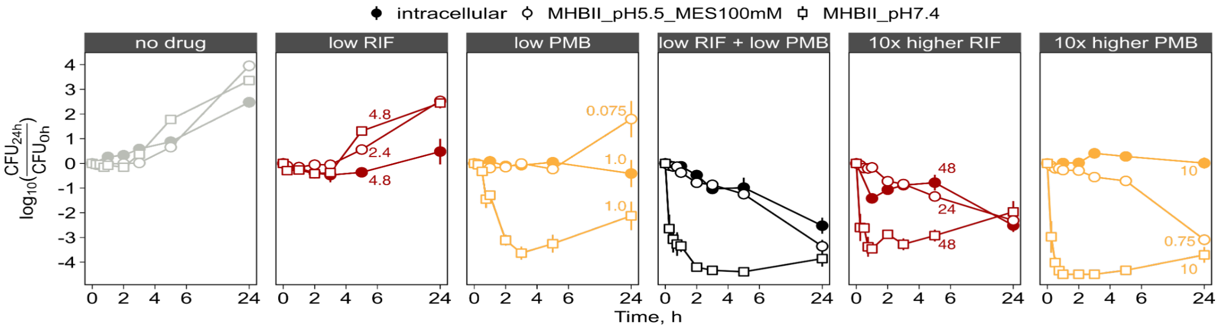

***P. aeruginosa* ATCC27853 growth and killing is more similar between intracellular and broth at pH 5.5 environments as opposed to broth at pH 7.4.** Bacteria were grown as described in **Figure S1A**. Then, antibiotics were added at the concentrations indicated in the figure, and their activity evaluated after sampling, serial dilution, and CFU counting. We used single drugs (low RIF and low PMB) at concentrations to give a similar 24 h efficacy in combination (low RIF + low PMB). In addition, we applied both drug monotherapies at 10x higher concentrations to reveal the similarities/discrepancies with the combination treatment. Exact concentrations in mg/L are indicated in the figure. Points are arithmetic means (4-7 biological replicates), and error bars are 95% CI.

Supplementary Figure 3

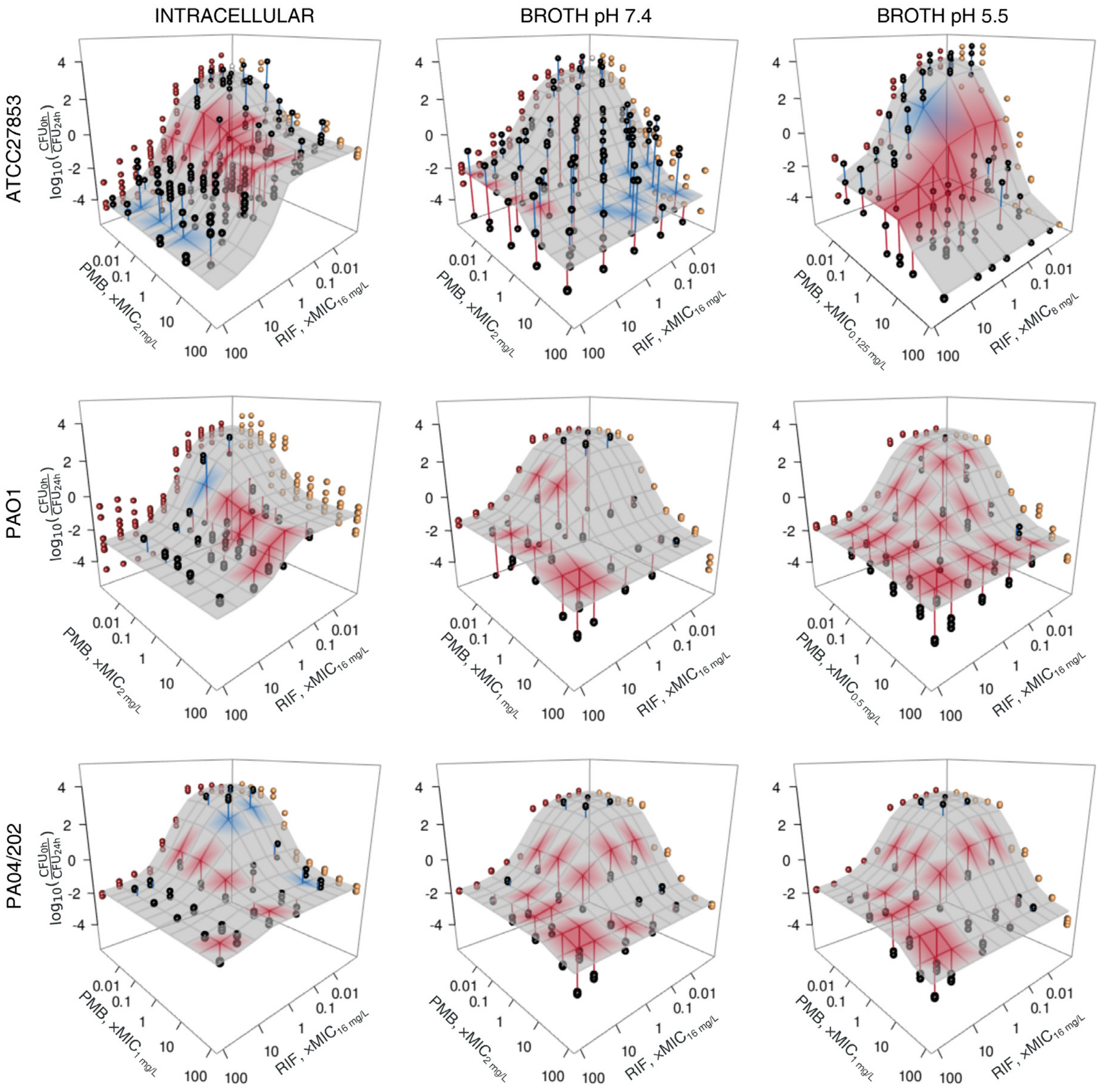

The combination is synergistic against all the *P. aeruginosa* strains. Synergy is most pronounced at 1x MIC of rifampicin in an intracellular experiment **(left column)**. While ATCC27853 has lost much of the synergy in broth at neutral pH, PAO1 and PA04/202 retained most of the synergy, albeit now it is less specific to 1x MIC of rifampicin **(middle column)**. Acidification of the broth **(right column)** brings back the synergy in ATCC27853 coupled with a substantial increase in polymyxin B potency (MIC dropped from 2 to 0.125 mg/L) and efficacy (-3.5 to -5.5 $\log_{10}$ of CFU/ml); acidification of broth makes little difference for PAO1 and PA04/202 in terms of synergy, though PAO1 aligns better with ATCC27853 for it has more sub-MIC synergy at low pH. Note that the results for ATCC27853 are the same as those in the figure in the main text, shown here just to facilitate comparison.

The experimentally measured outcome of the two-drug combination is shown with black circles (each circle represents a biological replicate of experiments performed in 3 technical replicates). The red lines signify synergy, i.e., connect points to the surface if the measured CFUs are below the expectation surface. Conversely, blue indicates antagonism. The surface was colored if the difference from expectation was statistically significant ($p < 0.05$ from a bootstrapped approximation of the expected result under Loewe's null).

Supplementary Figure 4

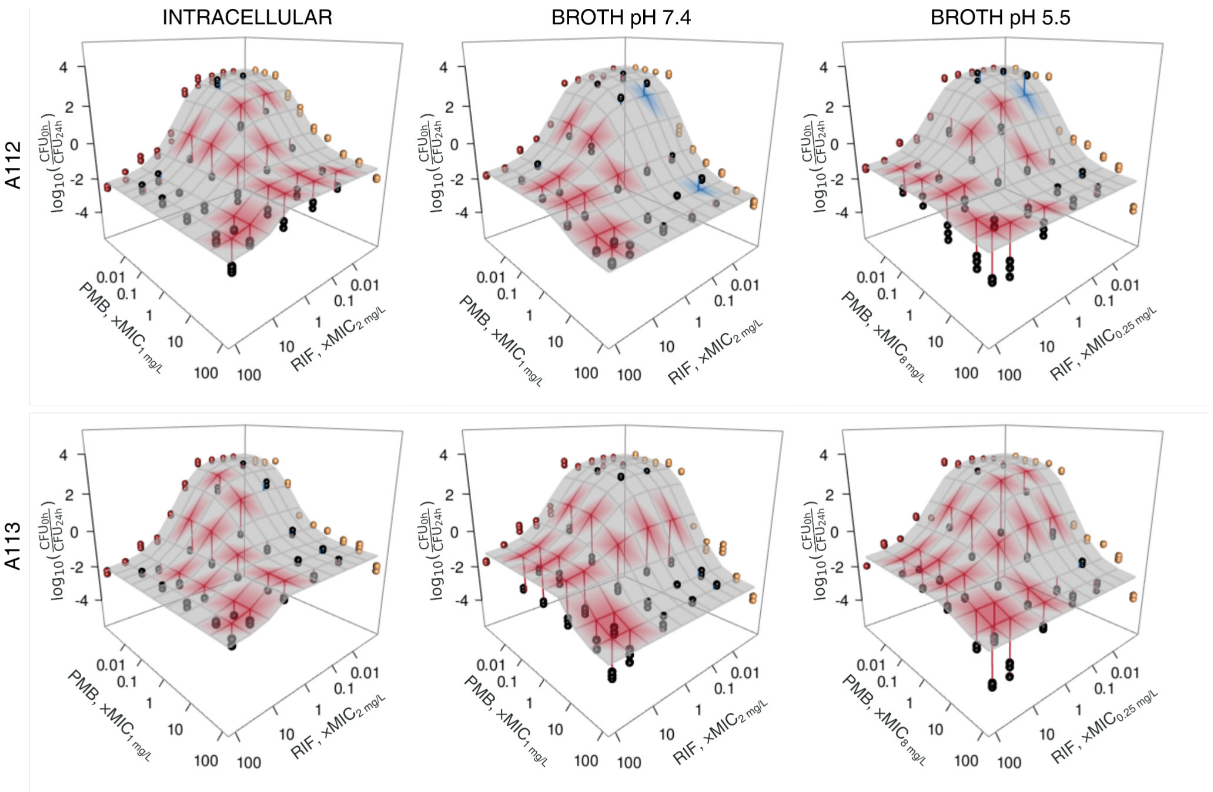

The combination is synergistic against *A. baumannii* clinical isolates. Synergy is most pronounced at 1x MIC of rifampicin in an intracellular experiment **(left column)**. Some of the synergy at sub-MIC rifampicin concentrations disappears in broth at neutral pH **(middle column)**, which acidification of the broth **(right column)** brings back. Compared to *P. aeruginosa*, rifampicin is eight times more potent against *A. baumannii* (intracellular and neutral broth, MIC 2 mg/mL), which is furthered to 64 times upon acidification (0.25 mg/L). Note that the intracellular results for isolate A112 are the same as those in the figure in the main text, shown here for completeness and to facilitate comparison. The experimentally measured outcome of the two-drug combination is shown with black circles (each circle represents a biological replicate of experiments performed in 3 technical replicates). The red lines signify synergy, i.e., connect points to the surface if the measured CFUs are below the expectation surface. Conversely, blue indicates antagonism. The surface was colored if the difference from expectation was statistically significant ($p < 0.05$ from a bootstrapped approximation of the expected result under Loewe's null).

Supplementary Figure 5

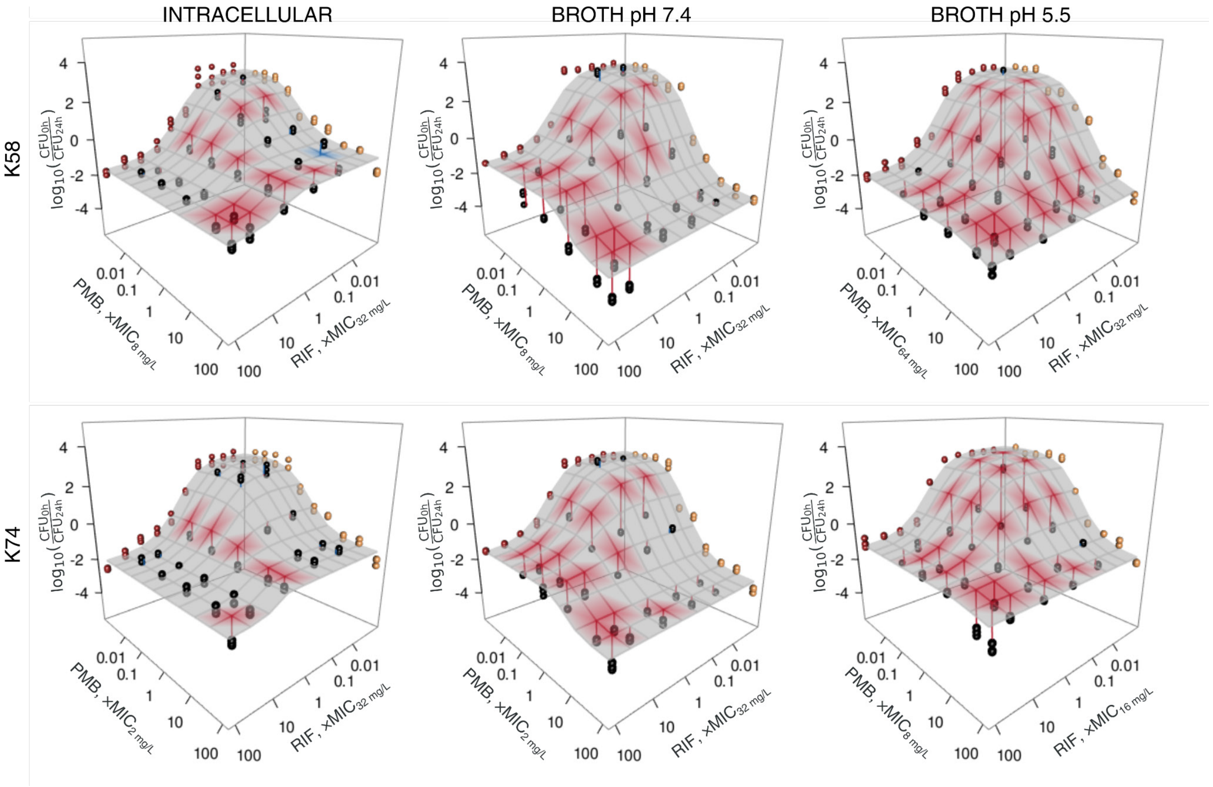

The combination is synergistic against *K. pneumoniae* clinical isolates. Both *K. pneumoniae* clinical strains show synergy in broth and intracellular experiments, which is more pronounced at 1x MIC of rifampicin **(left column)**. Synergy is even more prominent in broth at neutral pH (middle column). Acidification of the broth **(right column)** introduces additional synergy at sub-MIC rifampicin concentrations **(right column)**. Note that the intracellular results for isolate K58 are the same as in the figure in the main text, shown here for completeness and to facilitate comparison. The experimentally measured outcome of the two-drug combination is shown with black circles (each circle represents a biological replicate of experiments performed in 3 technical replicates). The red lines signify synergy, i.e., connect points to the surface if the measured CFUs are below the expectation surface. Conversely, blue indicates antagonism. The surface was colored if the difference from expectation was statistically significant ($p < 0.05$ from a bootstrapped approximation of the expected result under Loewe's null).

Supplementary Figure 6

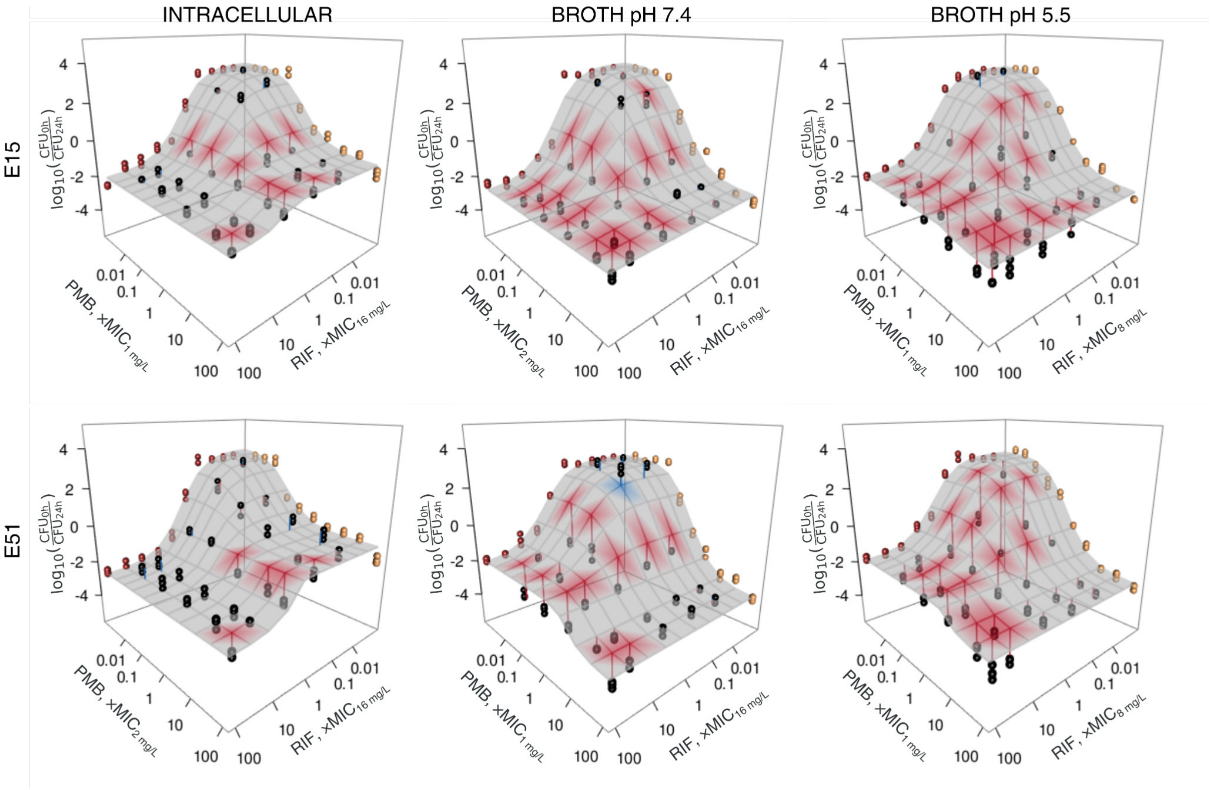

The combination is synergistic against *E. coli* clinical isolates. Synergy is most pronounced at 1x MIC of rifampicin in an intracellular experiment **(left column)**. Synergy is even more prominent in broth at neutral pH **(middle column)**. Acidification of the broth **(right column)** increases the number of synergistic concentrations and slightly the potency of drugs (within the prevalent two-fold variation in measurements of MIC). Note that the intracellular results for isolate E15 are the same as in the figure in the main text, shown here for completeness and to facilitate comparison. The experimentally measured outcome of the two-drug combination is shown with black circles (each circle represents a biological replicate of experiments performed in 3 technical replicates). The red lines signify synergy, i.e., connect points to the surface if the measured CFUs are below the expectation surface. Conversely, blue indicates antagonism. The surface was colored if the difference from expectation was statistically significant ($p < 0.05$ from a bootstrapped approximation of the expected result under Loewe's null).

Supplementary Figure 7

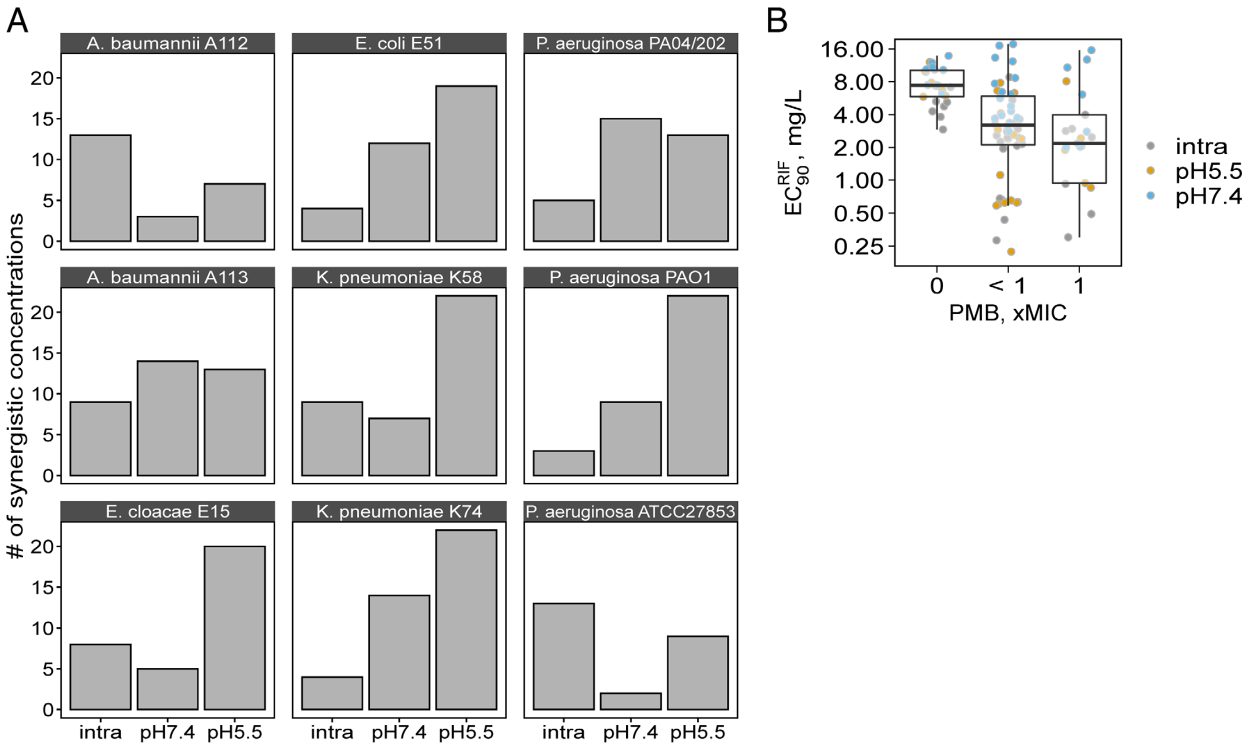

Acidification increases the number of drug concentration pairs with a synergistic effect. The number of significantly synergistic points for the results is summarised for results in **Figure S3**, **Figure S4**, **Figure S5**, and **Figure S6**.

Supplementary Figure 8

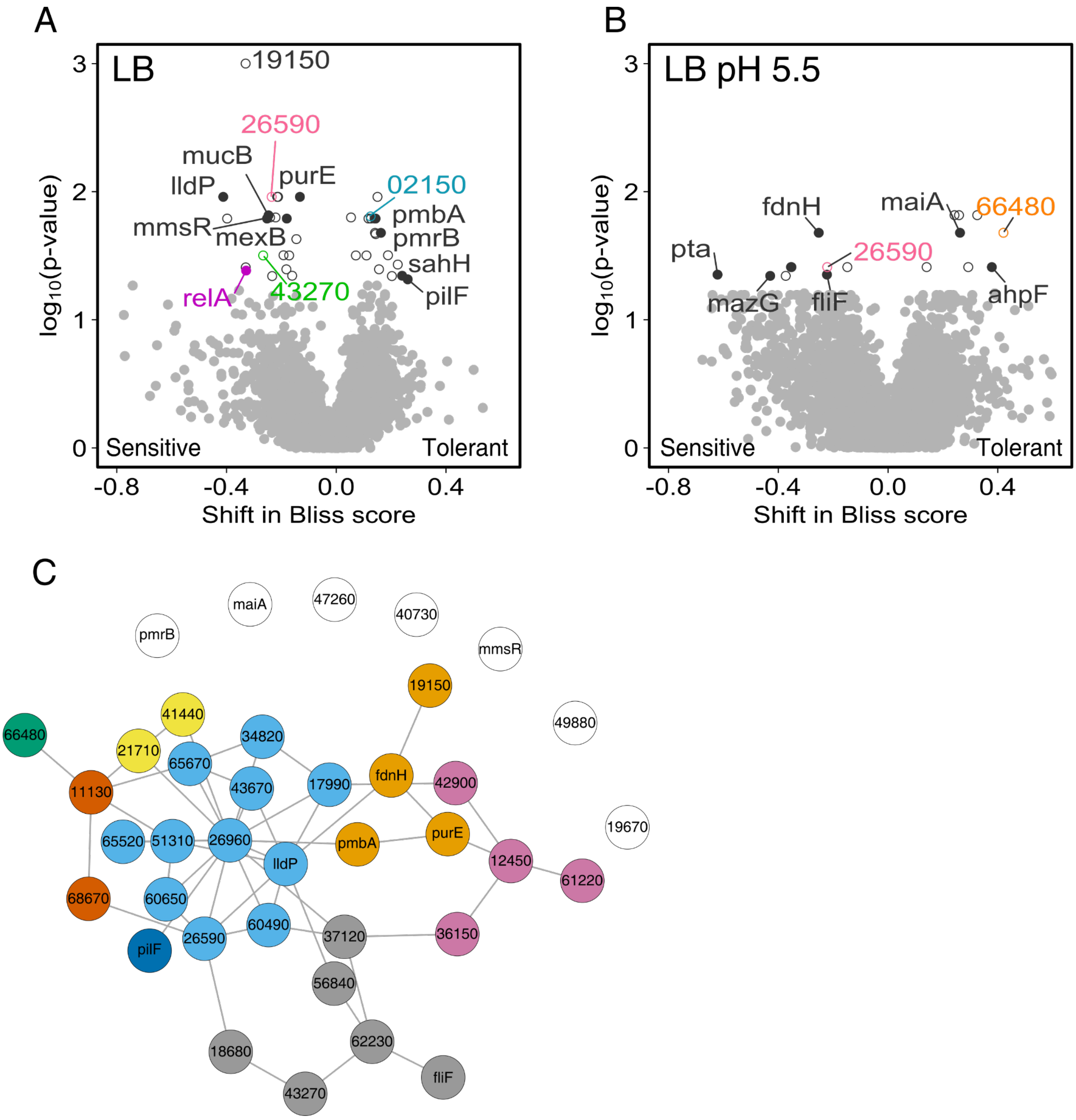

Results from chemical-genomics. (A, B) *P. aeruginosa* transposon mutants with altered sensitivity towards rifampicin–polymyxin B combination on solid LB (A) and LB at pH 5.5 (B). The genes in which the transposon resides, altered in their sensitivity towards the combination with a p-value less than 0.05, are drawn in black. Empty black figures are genes without names, for which only the ORF number is available. Coloured labels correspond to five mutants we interrogated after validating the hits (e.g. **Figure 3C-D** and **Figure S12**). Points are arithmetic mean (5 biological replicates). **(C)** The major graph communities of protein-protein interaction network of hits using Newman-Girvan's edge betweenness: 1) the light blue nodes are central and seem to be enriched in regulatory genes; 2) the grey nodes seem to be signal transduction from membrane to the regulatory genes (in light blue); 3) orange and pink nodes are mostly related to metabolism with those in orange having more membrane related terms than the pink nodes.

Supplementary Figure 9

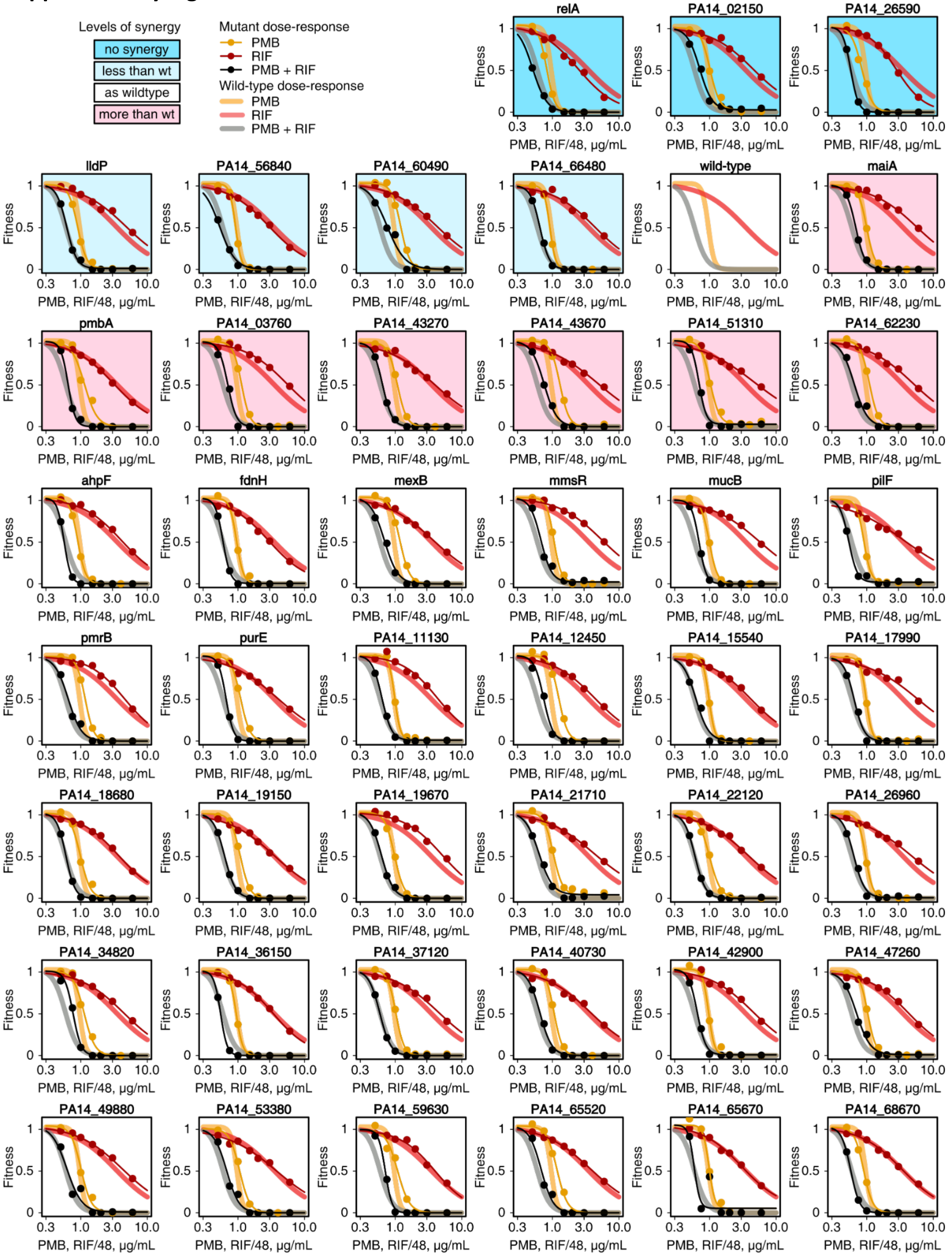

Concentration-response curves for *P. aeruginosa* PA14 mutants in comparison to wild-type. Points are an arithmetic mean (3-4 biological replicates), and curves are a fit with four-parameter logistic regression. For visualisation—to accommodate all the curves on the same plot—the rifampicin concentration is 48 times scaled-down (i.e. the actual rifampicin concentration was 48 times of what is indicated on the x-axis). SahH mutant, not shown, was growing so slowly that it needed a longer pre-culturing and eventually could not be analysed by Loewe's surface (**Figure S10**).

Supplementary Figure 10

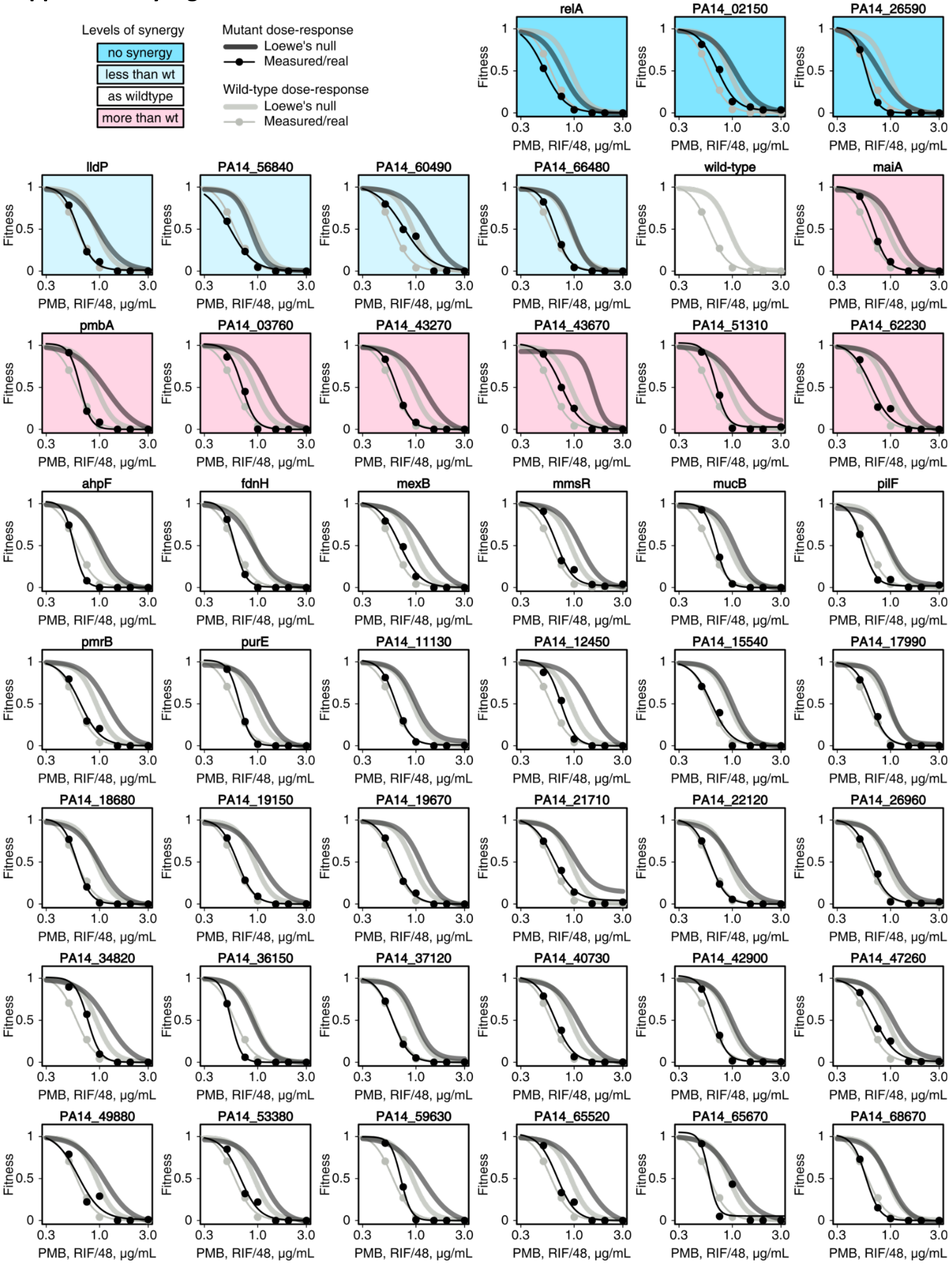

Dose-response curves of rifampicin–polymyxin B in comparison to expected results under Loewe's null. Measured/actual points are arithmetic means (3-4 biological replicates), and thin-line curves are four-parameter logistic regression. Under Loewe's null, the expectation was calculated from monotherapies (**Figure S10**). For visualisation—to accommodate all the curves on the same plot—the rifampicin concentration was 48 times scaled-down (i.e. the actual rifampicin concentration was always 48 times of what is indicated on the x-axis).

Supplementary Figure 11

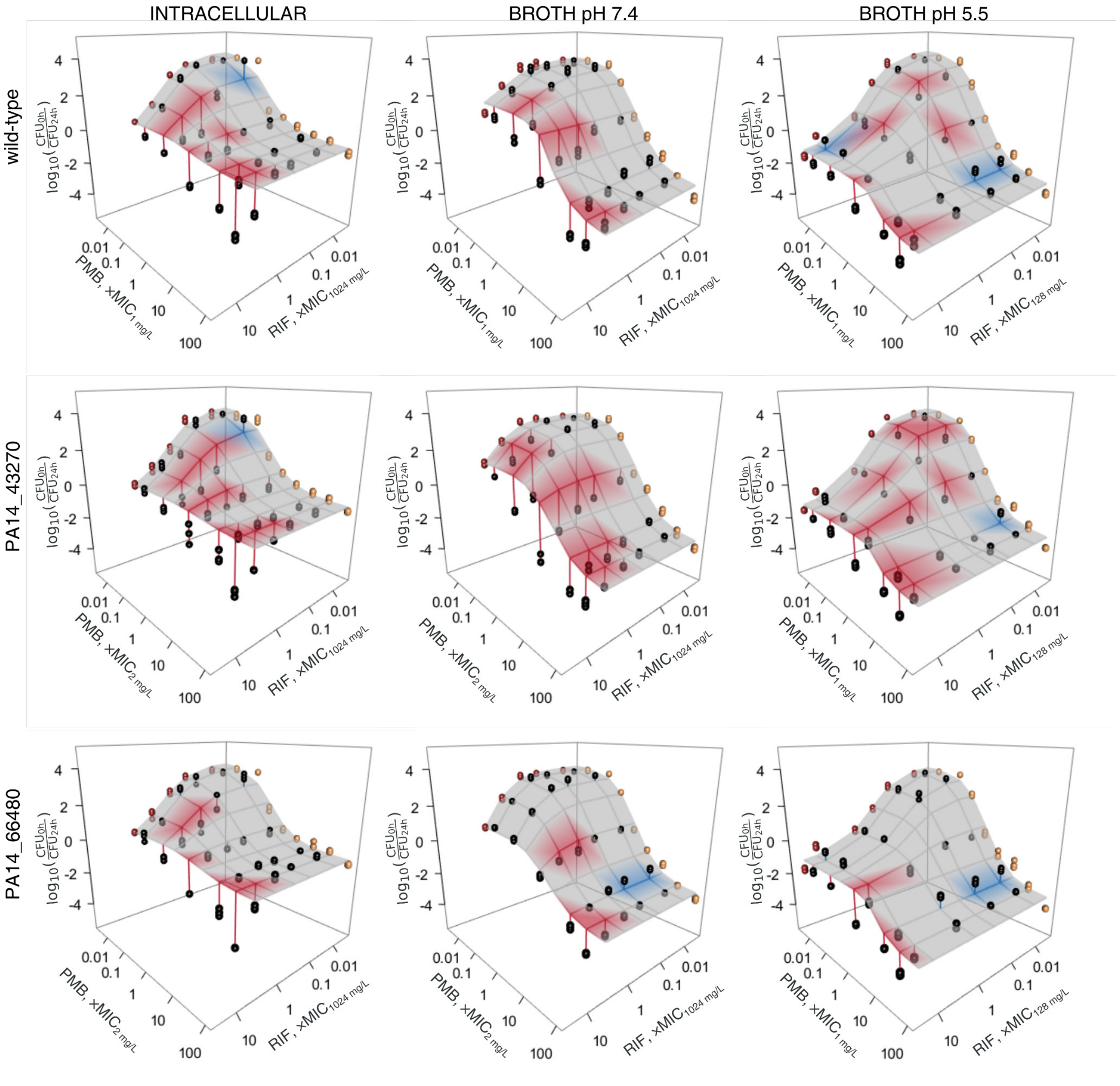

Selected transposon mutants have altered propensity towards rifampicin—polymyxin B interaction. Compared to isogenic wild-type *P. aeruginosa* PA14 Liberati **(top row)**, isogenic mutants with transposons in PA14_43270 **(middle row)** or PA14_66480 **(bottom row)** have more and fewer regions of synergy, respectively. The experimentally measured outcome of the two-drug combination is shown with black circles (each circle represents a biological replicate of experiments performed in 3 technical replicates). The red lines signify synergy, i.e., connect points to the surface if the measured CFUs are below the expectation surface. Conversely, blue indicates antagonism. The surface was colored if the difference from expectation was statistically significant ($p < 0.05$ from a bootstrapped approximation of the expected result under Loewe's null).

Supplementary Figure 12

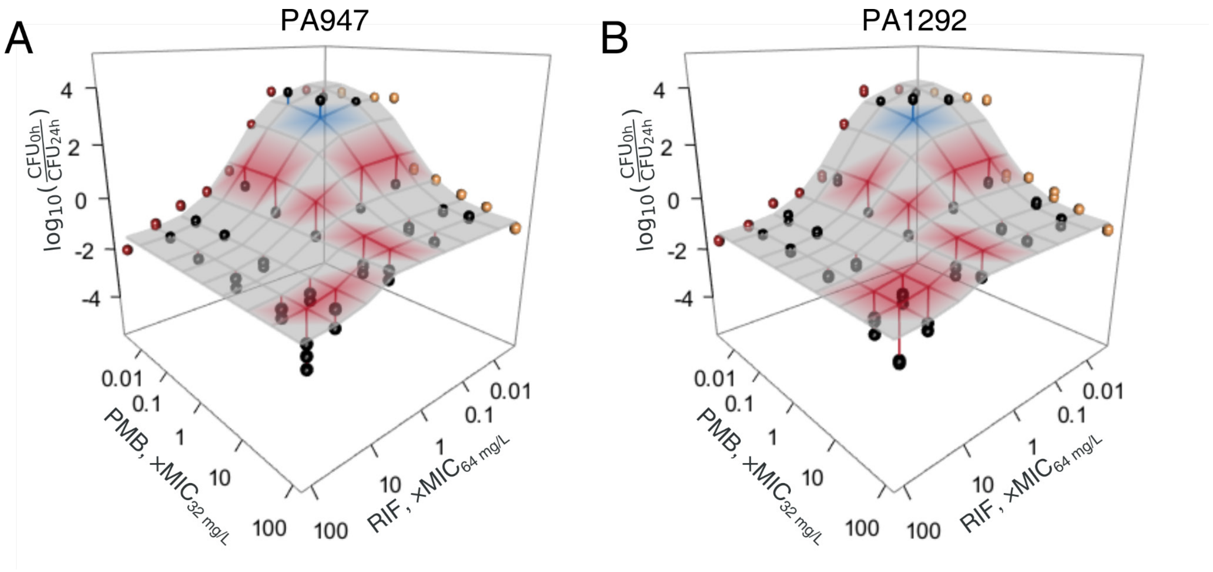

The combination is synergistic against intracellular polymyxin B resistant clinical isolates of *P. aeruginosa*. The experimentally measured outcome of the two-drug combination is shown with black circles (each circle represents a biological replicate of experiments performed in 3 technical replicates). The red lines signify synergy, i.e., connect points to the surface if the measured CFUs are below the expectation surface. Conversely, blue indicates antagonism. The surface was colored if the difference from expectation was statistically significant ($p < 0.05$ from a bootstrapped approximation of the expected result under Loewe's null).

Supplementary Figure 13

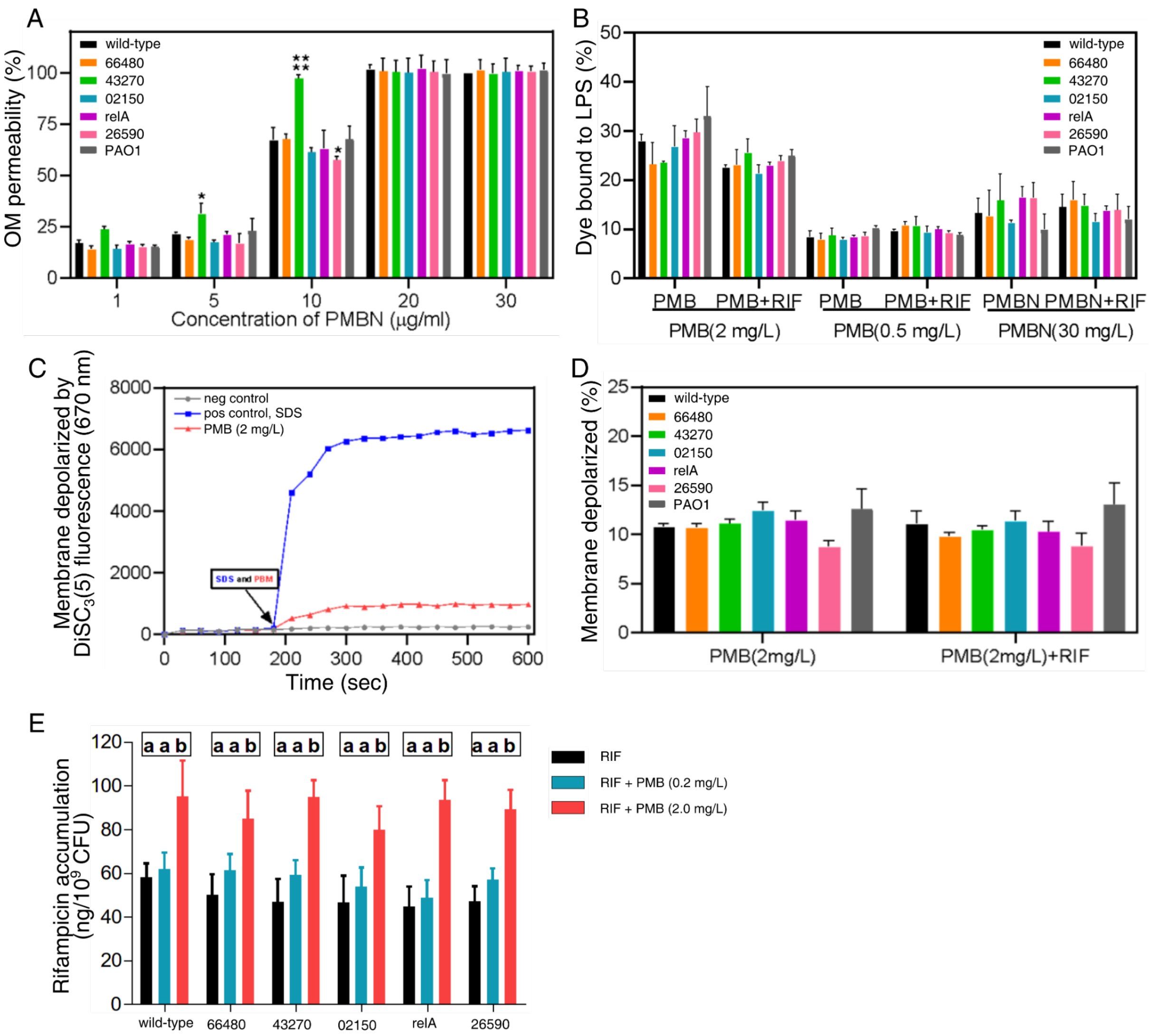

Altered membrane properties of the transposon mutants identified via chemical-genomics. (A) Outer membrane permeability upon increasing concentrations of polymyxin B nonapeptide (PMBN) for various *P. aeruginosa* PA14 strains and PAO1 for comparison. **(B)** BODIPY™-TR-cadaverine (BC) displacement assay for various *P. aeruginosa* PA14 strains and PAO1 for comparison. BC binds lipid A of LPS and, when displaced, becomes fluorescent (measured at $Ex_{580\ nm}$ and $Em_{620\ nm}$). Bacteria cells in buffer and 50 µM alexidine were a negative and positive control (100%), respectively. **(C)** Inner membrane depolarisation in time for various *P. aeruginosa* PA14 strains and PAO1 for comparison. Depolarisation was measured by $DiSC_3(5)$ fluorescence ($Em_{622\ nm}$, $Ex_{670\ nm}$). At 3 min, either SDS (0.5%) or PMB (2 mg/mL) was added. **(D)** Same as (C), membrane depolarisation is measured for various *P. aeruginosa* strains after 30 min of PMB or PMB + RIF treatment expressed as a percentage of SDS treatment. **(E)** The accumulation of rifampicin alone or combined with PMB in PA14 and its mutants. In 0.2 mg/L PMB (0.1 xMIC), there is no significant rifampicin accumulation increase in all isolates. In 2 mg/L PMB(1 xMIC), all isolates show a significant increase in rifampicin accumulation. Bars are the mean of three independent experiments performed in 3 replicates and error bars are SEM. Statistical analysis: (A), (B), (D) one-way ANOVA with Dunnet post-hoc test, p-values: * <0.5, **** <0.0001; (E) one-way ANOVA with a Tuckey post-hoc test (data series with different letters are different from one another, p < 0.05). Polymyxin B was used at concentrations indicated in the figure, and rifampicin was used at 16 mg/L.

Supplementary Table 3.

GO terms of a validated subset of genes.

PA gene	Location	Process	Function
02150	membrane	signal transduction	catalytic
03760	membrane	transmembrane transport	transmembrane transporter
26590	NA	regulation of transcription; biosynthesis	catalytic; transcription factor
43270	NA	tRNA seleno-modification	transferase for selenium-containing groups
43670	membrane	signal transduction; phosphorylation	phosphorelay sensor kinase
56840	NA	NA	electron transfer; heme binding
60490	NA	NA	electron transfer; heme binding
lldP	membrane	lactate transport	lactate transmembrane transport
maiA	cytoplasm	aromatic amino acid metabolism	catalytic; protein binding
pmbA	NA	peptidoglycan biosynthesis; proteolysis	metallopeptidase
relA	NA	ppGpp metabolic process	NA
